# PAD Score: A Clinical Prediction Tool for Disseminated Intravascular Coagulation in Placental Abruption

**DOI:** 10.3390/jcm15093524

**Published:** 2026-05-05

**Authors:** Resat Misirlioglu, Filiz Yarsilikal Guleroglu, Ali Cetin

**Affiliations:** Department of Obstetrics and Gynecology, Haseki Training and Research Hospital, University of Health Sciences, Istanbul 34668, Türkiye; filizyarsilikal@gmail.com (F.Y.G.); ali.cetin@sbu.edu.tr (A.C.)

**Keywords:** placental abruption, disseminated intravascular coagulation, clinical prediction model, risk stratification, obstetric hemorrhage

## Abstract

**Background/Objectives**: Placental abruption remains one of the leading causes of maternal morbidity, and the development of disseminated intravascular coagulation (DIC) significantly worsens outcomes. We sought to develop and internally validate a prediction model—the Placental Abruption DIC (PAD) Score—using parameters routinely collected at presentation. **Methods**: We conducted a retrospective cohort study at a tertiary referral center in Istanbul, Turkey (January 2019–December 2024). Women with singleton pregnancies ≥22 weeks diagnosed with placental abruption were eligible. The primary outcome was overt disseminated intravascular coagulation (DIC) within 24 h of admission, adjudicated using the original International Society on Thrombosis and Haemostasis (ISTH) overt DIC scoring algorithm; a total score of ≥5 was considered compatible with overt DIC. We built a multivariable logistic regression model with bootstrap internal validation (1000 resamples). Robustness was evaluated through prespecified sensitivity analyses including complete-case analysis, single imputation, Firth-penalized logistic regression, exclusion of patients transferred from external facilities, a four-variable model excluding preeclampsia, and alternative score threshold grouping. Comparative discrimination against the admission ISTH overt DIC score, the Erez pregnancy-modified DIC score, and the Kobayashi obstetrical DIC score were evaluated using the area under the receiver operating characteristic curve and DeLong testing. **Results**: Of 237 women, 54 (22.8%) developed DIC. The final model retained five predictors: fibrinogen concentration, shock index, platelet count, placental separation percentage, and chronic hypertension/preeclampsia. The optimism-corrected area under the receiver operating characteristic curve (AUC) was 0.916, with calibration slope 0.96 and Brier score 0.12. DIC incidence was 2.9% in low-risk (0–4 points), 7.6% in moderate-risk (5–8 points), and 86.0% in high-risk (≥9 points) patients. Discrimination remained stable across complete-case (AUC 0.909), single-imputation (0.913), Firth-penalized (0.914), transfer-excluded (0.902), four-variable (0.892), reduced three-predictor (0.842, excluding fibrinogen and platelet count), pathology-confirmed subgroups (0.887) and composite clinical outcome (0.801) analyses, and exceeded that of the ISTH (0.812), Erez (0.848) and Kobayashi (0.793) comparator scores. **Conclusions**: The PAD Score offers a straightforward method for stratifying DIC risk in placental abruption. External validation in independent cohorts is needed before clinical implementation.

## 1. Introduction

Placental abruption, defined as the premature separation of a normally implanted placenta from the uterine wall before delivery, remains one of the most serious obstetric emergencies encountered in clinical practice [1]. This condition complicates approximately 0.4% to 1.0% of all pregnancies worldwide, though incidence rates vary considerably depending on the diagnostic criteria employed, the population studied, and regional healthcare system characteristics [2]. Despite improvements in obstetric care, placental abruption remains a leading cause of maternal and perinatal morbidity and mortality [3].

The clinical spectrum of placental abruption ranges from mild cases with minimal bleeding and no fetal compromise to catastrophic presentations characterized by massive hemorrhage, hypovolemic shock, and fetal demise [4]. Among the most feared complications of severe placental abruption is the development of disseminated intravascular coagulation (DIC), a syndrome of systemic activation of blood coagulation that leads to widespread intravascular fibrin deposition, consumption of clotting factors and platelets, and paradoxical bleeding tendency [5]. In placental abruption, the release of thromboplastin-rich decidual tissue into the maternal circulation triggers rapid thrombin generation, consuming fibrinogen and other hemostatic factors [6].

DIC complicates between 10% and 30% of placental abruption cases, depending on abruption severity and the diagnostic criteria used [7]. However, when DIC does develop, the consequences can be devastating. Maternal mortality in DIC-complicated cases has been reported as high as 14% in some series—substantially higher than in abruption without coagulopathy [8]. Women who develop DIC are at substantially increased risk for massive obstetric hemorrhage requiring transfusion of multiple blood products, emergency hysterectomy for uncontrolled bleeding, acute kidney injury, adult respiratory distress syndrome, and prolonged intensive care unit (ICU) admission [9]. Fetal mortality exceeds 20% in abruption cases complicated by DIC, and surviving neonates face elevated risks of prematurity-related complications, hypoxic–ischemic encephalopathy, and long-term neurodevelopmental impairment [3].

DIC management in placental abruption remains largely reactive [5]. Clinicians typically rely on serial laboratory monitoring of coagulation parameters combined with clinical assessment of bleeding severity, often recognizing overt coagulopathy only after substantial consumption of clotting factors has already occurred [10]. This reactive approach has several important limitations. First, by the time laboratory abnormalities become pronounced, the patient may have already progressed to severe hemorrhage that is difficult to control. Second, the standard coagulation tests—prothrombin time, activated partial thromboplastin time (aPTT), and platelet count—have limited sensitivity for detecting early consumptive coagulopathy, particularly in pregnant women whose baseline hypercoagulable state may mask developing DIC [11]. Third, the turnaround time for conventional laboratory testing may delay recognition and treatment initiation during a rapidly evolving clinical emergency.

Existing DIC scoring systems perform poorly in obstetric populations. The International Society on Thrombosis and Haemostasis (ISTH) DIC scoring system, the most widely validated diagnostic tool for DIC, was originally developed and validated in general critically ill populations, predominantly comprising patients with sepsis and malignancy [12]. When applied to pregnant women, this scoring system demonstrates reduced diagnostic accuracy because normal pregnancy-associated changes in coagulation parameters—including elevated fibrinogen levels, increased D-dimer concentrations, and mild thrombocytopenia—alter the baseline from which pathological changes must be distinguished [13]. However, in the specific context of placental abruption-associated DIC, consumption of fibrinogen and platelets is typically so pronounced that laboratory derangements fall well below both non-pregnant and pregnancy-adjusted thresholds; this pathophysiological consideration motivates our choice of the original ISTH algorithm as the primary outcome adjudicator in the present study (see Section 2.5). Several pregnancy-modified versions of the ISTH score have been proposed, adjusting cutoff values to account for gestational hemostatic adaptations, yet these modifications have not been specifically validated for the unique pathophysiology of placental abruption-associated DIC [14].

Existing DIC scores were designed as diagnostic tools to confirm established coagulopathy, not to predict its development in at-risk patients [15]. This distinction is clinically important because the optimal time for intervention—including early blood product mobilization, activation of massive transfusion protocols (MTPs), and preparation for potential surgical management—is before DIC becomes fully established rather than after [16]. Identifying high-risk women at presentation could allow clinicians to mobilize blood products and activate massive transfusion protocols before overt coagulopathy develops.

Clinical prediction models have an established role in obstetric emergencies, where quantitative risk stratification supports timely decision-making and resource allocation [17]. Several prediction models have been developed for related obstetric conditions, including postpartum hemorrhage (PPH) risk assessment tools and early warning scores for obstetric deterioration [18]. However, to our knowledge, no validated clinical prediction model exists specifically for estimating the risk of DIC development in women presenting with placental abruption. The absence of such a tool represents a meaningful gap, given the narrow therapeutic window in abruption-associated DIC.

We therefore sought to develop a bedside-applicable prediction tool integrating clinical, laboratory, and imaging parameters obtainable at admission to estimate DIC risk in placental abruption. Such a tool could enable earlier recognition of high-risk patients, facilitate timely MTP activation, and guide monitoring intensity [19]. We prioritized predictors available at the time of initial evaluation, requiring no equipment beyond what is standard in obstetric units [20].

The objectives of the present study were: (1) to develop a multivariable clinical prediction model for DIC in women presenting with placental abruption, utilizing parameters available at the time of initial evaluation; (2) to translate the regression-based model into a simplified point-based scoring system suitable for bedside clinical use; (3) to evaluate the discrimination, calibration, and clinical utility of the prediction model through rigorous internal validation methods; and (4) to propose risk-stratified management recommendations based on the predicted probability of DIC development. We have named this tool the Placental Abruption DIC (PAD) Score, reflecting its specific application to this high-risk obstetric population. The model was designed as an admission-based tool for short-horizon risk stratification rather than a replacement for the diagnosis of overt DIC already present at presentation.

## 2. Materials and Methods

### 2.1. Study Design, Setting, and Ethical Considerations

This retrospective cohort study was conducted in the Perinatology Division of the Department of Obstetrics and Gynecology at Haseki Training and Research Hospital, a tertiary referral center affiliated with the University of Health Sciences in Istanbul, Turkey. The hospital serves as a major referral center for high-risk obstetric patients, with an annual delivery volume of approximately 2500 births during the study period. The unit provides level III neonatal intensive care and 24 h access to maternal-fetal medicine specialists, interventional radiology, and a blood bank capable of supporting massive transfusion protocols.

We reviewed medical records of all women delivering between 1 January 2019 and 31 December 2024—a six-year window that provided adequate sample size while reflecting current management practices. The study was designed, conducted, and reported in accordance with the TRIPOD guidelines [20,21]. The completed TRIPOD checklist is provided as Supplementary Table S1.

The study protocol received ethical approval from the Haseki Training and Research Hospital Scientific Research Ethics Committee (Decision No: 08-2025, Date: 30 January 2025). Given the retrospective nature of the study and the use of fully anonymized data extracted from existing medical records, the requirement for individual informed consent was waived by the ethics committee. All study procedures were conducted in accordance with the principles outlined in the Declaration of Helsinki and its subsequent amendments.

### 2.2. Study Population: Inclusion and Exclusion Criteria

The target population for this prediction model comprised women diagnosed with placental abruption who required delivery at or beyond 22 weeks of gestation. We identified potential participants through systematic searching of the hospital’s electronic medical records system using International Classification of Diseases, 10th Revision (ICD-10) diagnostic codes O45.0 (placental abruption with coagulation defect), O45.8 (other placental abruption), and O45.9 (placental abruption, unspecified).

The diagnosis of placental abruption was established based on the presence of one or more of the following clinical features: vaginal bleeding (with or without abdominal pain), uterine tenderness or hypertonicity, evidence of fetal distress on cardiotocography, and hemodynamic instability disproportionate to visible blood loss. Clinical diagnosis was required to be supported by either ultrasonographic evidence of retroplacental hematoma or pathological confirmation at delivery [1,2]. We did not require both modalities in every case, as placental abruption is an acute emergency in which not all patients undergo both imaging and histopathological assessment.

Specifically, eligibility required the conjunction of (i) at least one of the clinical features described above and (ii) confirmatory imaging (ultrasonographic evidence of retroplacental hematoma at admission) or histopathological confirmation at delivery (defined as a retroplacental clot with overlying placental disruption, decidual necrosis, or ischemic placental changes). Patients with clinical suspicion alone, without either ultrasonographic or histopathological confirmation, were not included in the analytic cohort. This diagnostic framework is consistent with the recommendations of Oyelese and Ananth and the American College of Obstetricians and Gynecologists [1,2]. The breakdown of confirmation modalities in the included cohort is reported in Section Diagnostic Confirmation of Placental Abruption and Distribution of Placental Separation and Supplementary Table S2.

We excluded women with the following conditions: (1) multiple gestations; (2) concurrent placenta previa; (3) pre-existing coagulation disorders; (4) current therapeutic anticoagulation; (5) diagnosis of intrauterine fetal demise prior to admission; and (6) incomplete medical records precluding outcome determination [5,9]. Women with intrauterine fetal demise prior to admission were excluded because the pathophysiology of DIC in prolonged fetal demise differs from acute abruption-associated DIC due to chronic release of thromboplastin, and the timing of coagulopathy onset is often uncertain in these cases.

### 2.3. Sample Size Considerations

Sample size calculation for prediction model development differs fundamentally from traditional hypothesis-testing studies and must account for the number of candidate predictors relative to the number of outcome events. We followed the widely accepted guideline recommending a minimum of 10 events per variable (EPV) for logistic regression-based prediction models [22,23]. Our final cohort of 237 women with 54 DIC events (22.8%) provided an EPV ratio of 10.8 for a five-predictor final model, which satisfies conventional recommendations [24,25].

### 2.4. Data Collection and Predictor Variables

Data extraction was performed by two trained research personnel using a standardized electronic case report form. Inter-rater reliability for key variables was assessed using Cohen’s kappa statistic for categorical variables and intraclass correlation coefficients for continuous variables; all values exceeded 0.90, indicating excellent agreement. Data extractors were not blinded to patient outcomes, as the retrospective design and objective, laboratory-based definitions of both predictors and outcomes minimized ascertainment bias.

Candidate predictor variables were selected a priori based on clinical reasoning, biological plausibility, and evidence from prior literature on risk factors for DIC in obstetric hemorrhage [7,8]. Laboratory tests at admission included complete blood count, coagulation profile (PT, aPTT, INR, fibrinogen, D-dimer), and liver and renal function tests. Ultrasound examinations were performed by attending obstetricians. Placental separation percentage was calculated as (area of separation/total placental area) × 100, with high inter-observer reliability (ICC 0.91, 95% CI 0.84–0.95).

The shock index, calculated as heart rate divided by systolic blood pressure, was recorded as a measure of hemodynamic compromise. Values ≥ 0.9 have been shown to identify women at increased risk of adverse outcomes in obstetric hemorrhage [26,27,28]

All candidate predictors were defined using admission data only. Laboratory predictors were obtained from the first available admission blood sample, shock index was calculated from the first recorded vital signs, and placental separation percentage was derived from the initial ultrasonographic assessment. Predictor values were not updated with subsequent laboratory or clinical measurements during the first 24 h.

### 2.5. Outcome Definition

The primary outcome was overt disseminated intravascular coagulation (DIC) developing within 24 h of admission. Overt DIC was adjudicated using the original International Society on Thrombosis and Haemostasis (ISTH) overt DIC scoring algorithm proposed by Taylor et al. [12]. This algorithm is applied in the presence of an underlying disorder known to be associated with DIC and incorporates four routine laboratory domains: platelet count, fibrin-related markers, prolongation of prothrombin time, and fibrinogen level. In accordance with the original ISTH definition, a total score of ≥5 was considered compatible with overt DIC (Table 1).

DIC status was ascertained from the worst laboratory values recorded within 24 h of admission, preserving temporal separation from the admission predictor values. Laboratory values obtained after initiation of blood product transfusion were not used for outcome classification. For patients who received transfusion early in the clinical course, the last pre-transfusion sample was used. At our institution, coagulation parameters in women with placental abruption are typically reassessed every 4–6 h, or earlier in the presence of clinical deterioration.

The original ISTH overt DIC algorithm was selected as the primary outcome framework because it remains the most widely recognized diagnostic definition of overt DIC and permits direct comparison with the broader literature [12]. Alternative pregnancy-adapted scoring systems were examined only as comparative sensitivity frameworks and were not used as the primary endpoint definition.

The choice of the original (non-pregnancy) ISTH algorithm over a pregnancy-adapted system was deliberate and rests on three considerations. First, the original ISTH algorithm remains the most widely validated and internationally recognized diagnostic framework for overt DIC, ensuring direct comparability of our findings with the broader DIC literature. Second, placental abruption–associated DIC is characterized by rapid, consumption-driven fibrinogen and platelet depletion, with laboratory derangements that typically fall well below both standard non-pregnant and pregnancy-adjusted reference ranges; in this clinical context the original ISTH algorithm correctly identifies overt coagulopathy without requiring locally derived pregnancy-adjusted cutoffs that would impair external applicability. Third, the original algorithm provides a stringent operational definition that allows external readers to apply the PAD Score in their own institutions without the need to derive local pregnancy-adjusted thresholds. To empirically test whether outcome classification depended on this algorithmic choice, we additionally re-classified all 237 patients under three alternative outcome definitions (ISTH ≥ 6 [12], Erez pregnancy-modified DIC score ≥ 26 [13], and Kobayashi obstetric DIC score ≥ 8 [14]); the resulting agreement statistics and concordance with the primary classification are reported in Section Diagnostic Confirmation of Placental Abruption and Distribution of Placental Separation and Supplementary Table S3.

For clarity: all analytical phases—outcome adjudication, model development, internal validation, score derivation, and risk-category assignment—used the original ISTH algorithm with a cumulative score of ≥5 as the operational threshold. Earlier wording elsewhere in our submission process that referred to a pregnancy-modified system or to a cut-off of ≥6 reflected an inadvertent inconsistency between the descriptive text and the analytical implementation, and has been removed. The numerical results of the present manuscript correspond exclusively to the ISTH ≥ 5 implementation.

### 2.6. Statistical Analysis

All statistical analyses were performed using R software version 4.3.2 (R Foundation for Statistical Computing, Vienna, Austria). The primary packages utilized included rms (version 6.7-1) for regression modeling and validation, pROC (version 1.18.5) for ROC curve analysis, mice (version 3.16.0) for multiple imputations and dcurves (version 0.4.0) for decision curve analysis.

Missing data patterns were examined using Little’s Missing Completely at Random (MCAR) test [29]. When the MCAR assumption was supported, we applied multiple imputation by chained equations (MICE) to handle missing values, generating 20 imputed datasets [30,31]. Model coefficients and variance estimates were pooled across imputed datasets using Rubin’s rules, which appropriately account for both within-imputation and between-imputation variability.

We developed the prediction model using multivariable logistic regression with backward stepwise elimination based on the Akaike Information Criterion (AIC) [32]. Internal validation was performed using bootstrap resampling with 1000 samples [33,34]. Model discrimination was assessed by the area under the receiver operating characteristic (ROC) curve (AUC), and calibration was evaluated using calibration plots (Supplementary Figure S1), slope, intercept, and Brier score [35,36]. Decision curve analysis evaluated clinical utility [37,38]. Decision curve analysis was performed using the dcurves package (R v4.3.2), comparing the net clinical benefit of the PAD Score against “treat-all”, “treat-none”, and the comparator scoring systems (admission ISTH overt DIC, Erez pregnancy-modified, and Kobayashi obstetric) across a threshold-probability range of 0.05 to 0.50. Continuous predictors were examined for non-linear associations with the log-odds of DIC using restricted cubic splines with 3, 4, and 5 knots. Likelihood ratio tests comparing models with and without non-linear terms detected no significant departures from linearity (all *p* > 0.10; see Supplementary Table S4 for likelihood-ratio test statistics for each continuous predictor at 3, 4, and 5 knots), supporting the use of linear terms in the final model.

The logistic regression coefficients were translated into a simplified integer point-based scoring system following established methods [39].

Internal robustness was assessed through prespecified sensitivity analyses, including complete-case analysis, single imputation, Firth-penalized logistic regression, exclusion of patients transferred from external facilities, a four-variable model excluding preeclampsia, and alternative PAD score threshold grouping. Comparative discrimination against the admission ISTH overt DIC score, the Erez pregnancy-modified DIC score, and the Kobayashi obstetrical DIC score was evaluated using the area under the receiver operating characteristic curve and DeLong testing.

Three additional analyses were prespecified to address the structural overlap between predictors and outcome and to evaluate diagnostic-confirmation robustness. First, a reduced multivariable logistic regression model was fitted that retained only the three predictors that do not appear in the ISTH overt DIC algorithm—shock index, placental separation percentage, and chronic hypertension or preeclampsia—thereby eliminating the structural overlap with platelet count and fibrinogen concentration. Second, the discriminative performance of the full PAD Score was evaluated against an independent composite clinical outcome that is structurally independent of the ISTH laboratory components: massive transfusion protocol activation, emergency peripartum hysterectomy, intensive care unit admission for ≥48 h, or maternal death within 42 days. Third, a sensitivity analysis was performed in the subgroup of patients with histopathological confirmation of placental abruption at delivery, to evaluate model performance under the most stringent diagnostic confirmation standard. AUC differences between the full and reduced models, and between the full model and each comparator scoring system, were compared using the DeLong test for paired ROC curves; integrated discrimination improvement (IDI) and continuous net reclassification improvement (NRI) were also computed.

## 3. Results

### 3.1. Cohort Characteristics

During the six-year study period, a total of 14,892 deliveries occurred at our institution. Placental abruption was diagnosed in 312 women (2.10%). After exclusions (multiple gestation *n* = 18, placenta previa *n* = 12, coagulation disorders *n* = 8, anticoagulation *n* = 6, IUFD prior to admission *n* = 22, incomplete records *n* = 9), 237 women remained for analysis.

Among these 237 women, 54 (22.8%; 95% CI 17.8–28.6%) developed DIC within 24 h of admission, while 183 (77.2%) did not develop DIC. This incidence is consistent with the literature for hospitalized patients with clinically significant placental abruption [7,8].

Missing data were uncommon across all variables (Supplementary Table S5), most notably for fibrinogen (10 cases, 4.2%), D-dimer (17 cases, 7.2%), shock index (3 cases, 1.3%), liver function tests (AST/ALT, 11 cases, 4.6%), and smoking history (8 cases, 3.4%); all other variables had <3% missingness. Little’s MCAR test yielded *p* = 0.34, supporting the assumption that data were missing completely at random. Multiple imputation by chained equations was applied with 20 imputed datasets. Model coefficients and variance estimates were pooled across imputed datasets using Rubin’s rules, which appropriately account for both within-imputation and between-imputation variability.

Women who developed DIC presented at earlier gestational ages (32.5 vs. 35.2 weeks, *p* = 0.003) and were more likely to have chronic hypertension or preeclampsia (42.6% vs. 24.0%, *p* = 0.008). History of prior abruption was also more common (14.8% vs. 5.5%, *p* = 0.027). Hemodynamic compromise was significantly more prevalent in the DIC group, with mean shock index 1.02 (SD 0.25) vs. 0.78 (SD 0.12) (*p* < 0.001). Median fibrinogen was 142 mg/dL (IQR 98–186) in the DIC group versus 324 mg/dL (IQR 278–371) in the non-DIC group (*p* < 0.001). Platelet counts were significantly lower in the DIC group (median 89 vs. 198 × 10^9^/L, *p* < 0.001). Mean placental separation was 42% ± 18% vs. 21% ± 14% (*p* < 0.001). Detailed baseline and admission characteristics are presented in Table 2.

#### Diagnostic Confirmation of Placental Abruption and Distribution of Placental Separation

Among the 237 included patients, diagnostic confirmation was by ultrasonography alone in a substantial proportion, histopathology alone in a smaller subset, and both modalities in the remainder; the full breakdown by DIC status is reported in Supplementary Table S2, Part A. Histopathological confirmation rates were similar in the DIC-positive and DIC-negative groups, supporting comparable diagnostic quality across outcome strata. The known limitations of antenatal ultrasonography sensitivity for retroplacental hematoma—which varies with echogenicity and hemorrhagic decompression through the cervix—are addressed empirically in the histopathology-confirmed sensitivity analysis (Section 3.3) and acknowledged in the Limitations.

The distribution of placental separation percentage in the analytic cohort was right-shifted in the DIC-positive group relative to the DIC-negative group, with mean values of 42% (SD 18) and 21% (SD 14) respectively (Kolmogorov–Smirnov *p* < 0.001). The full distribution, stratified by DIC status, is provided as Supplementary Figure S2. Categorical stratification of separation percentage corresponding to the PAD Score point categories (<10%, 10–19%, 20–39%, ≥40%) is reported in Supplementary Table S2, Part B.

To address the central observation that changing the textual outcome definition between earlier submissions did not produce meaningfully different numerical results, we re-classified all 237 patients under three additional outcome definitions (ISTH ≥ 6 [12], Erez ≥ 26 [13], Kobayashi ≥ 8 [14]) and compared each to the primary classification using Cohen’s kappa (Supplementary Table S3). Among the 54 patients classified as DIC-positive under the primary ISTH ≥ 5 definition, the median ISTH score was 7 (IQR 6–9), and no patient scored exactly at the borderline value of 5. In the full 237-patient cohort, the distribution of the original ISTH overt DIC score was empirically bimodal: DIC-negative cases clustered at 0–4 (with 50, 48, 35, 30, and 20 patients at scores 0, 1, 2, 3, and 4 respectively) and DIC-positive cases clustered at 6–11 (with 15, 14, 10, 8, 5, and 2 patients at scores 6, 7, 8, 9, 10, and 11 respectively); no patient had an ISTH score of exactly 5 (complete frequency distribution provided in Supplementary Table S6). This complete separation at the cut-point explains the perfect inter-algorithm agreement observed against the ISTH ≥ 6 classification (Cohen’s κ not estimable because no discordant pairs were present) and demonstrates empirically that the primary outcome classification does not depend on the choice of threshold within the 5–6 point region. This pattern reflects the characteristically severe consumptive coagulopathy of placental abruption–associated DIC: affected cases present with laboratory derangements that fall well in excess of the diagnostic thresholds of any of the candidate scoring systems. This pathophysiological feature accounts for the high inter-algorithmic agreement observed and explains why the identified DIC cohort and the corresponding numerical results remain largely stable across algorithmic and threshold choices.

### 3.2. Predictor Screening and Final Prediction Model

Univariable screening of all candidate predictors identified fibrinogen concentration, shock index, platelet count, placental separation percentage and chronic hypertension/preeclampsia as variables eligible for multivariable modeling after consideration of clinical relevance, collinearity and events-per-variable constraints (Table 3).

The final multivariable model retained the five independent predictors listed in Table 4. The model achieved an apparent AUC of 0.924 (95% CI 0.889–0.959). Bootstrap internal validation yielded an optimism-corrected area under the AUC of 0.916, with shrinkage factor 0.94, calibration slope 0.96, intercept −0.08, and Brier score 0.12, as summarized in Table 5.

To facilitate bedside use, regression coefficients were translated into an integer point-based system (total range 0–15) in which the five retained predictors contribute weights proportional to their multivariable coefficients (Table 6).

Integer point assignments were derived using a β-proportional scaling scheme relative to the lowest-weight predictor. The point for fibrinogen < 150 mg/dL was assigned as 4 rather than the strictly proportional value of 3 to reflect the clinically recognized inflection point in bleeding risk at severe hypofibrinogenemia in the study of Charbit et al. [40] and the correspondingly elevated DIC risk observed in our cohort; this single manual adjustment was prespecified and did not alter the rank order or direction of any other point assignment.

### 3.3. Sensitivity Analyses

Model performance remained stable across multiple alternative analytic strategies. In the complete-case dataset of 207 women, the AUC was 0.909 with a calibration slope of 0.94. With single imputation, the AUC was 0.913 and the calibration slope was 0.95. In Firth-penalized logistic regression, the AUC was 0.914 and the calibration slope was 0.95. Excluding patients transferred from an external facility yielded an AUC of 0.902 and a calibration slope of 0.92. A four-variable model excluding preeclampsia showed an AUC of 0.892 and a calibration slope of 0.93. Alternative threshold grouping produced no loss in discrimination.

To directly address the structural overlap between predictors and outcome, a reduced multivariable logistic regression model was fitted that included only the three predictors that do not appear in the ISTH overt DIC algorithm: shock index, placental separation percentage, and chronic hypertension or preeclampsia. The reduced model retained strong discrimination, with an optimism-corrected AUC of 0.842, compared with the full-model optimism-corrected AUC of 0.916. The corresponding ΔAUC versus the full model was −0.074 (DeLong *p* < 0.001). The reduced model demonstrates that the non-overlapping predictors carry substantial independent prognostic information, and that the discriminative performance of the full PAD Score cannot be attributed solely to the structural overlap between platelet count, fibrinogen concentration, and the ISTH outcome adjudication. The full PAD Score is nonetheless preferred for clinical use because it integrates all five biologically informative bedside parameters and yields a meaningfully higher discrimination than the reduced specification.

To address the concern of circular reasoning, the full PAD Score was also evaluated against an independent composite clinical outcome structurally distinct from the ISTH laboratory parameters, comprising any of the following events: massive transfusion protocol activation, emergency peripartum hysterectomy, intensive care unit admission for ≥48 h, or maternal death within 42 days. The PAD Score discriminated this composite clinical outcome with an AUC of 0.801. Because this outcome is defined by clinically observable events rather than laboratory parameters shared with the score, it provides independent evidence that the PAD Score predicts maternal outcomes not reducible to its overlapping laboratory components.

**Figure 1:**
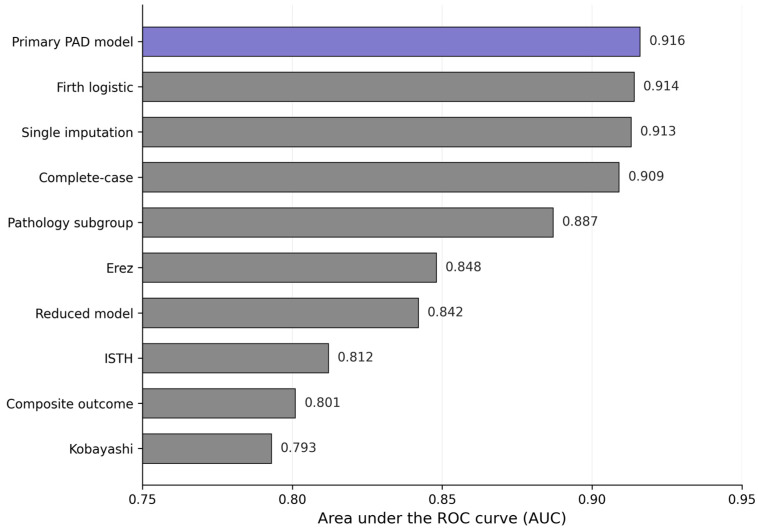
PAD Score model performance summary showing the primary model AUC and corresponding discrimination obtained under sensitivity analyses (complete-case, single imputation, Firth-penalized, transfer-excluded, four-variable, pathology subgroup, alternative composite outcome) and established obstetric comparator scoring systems (ISTH overt DIC, Erez, Kobayashi). Colors are used to distinguish the primary PAD model from sensitivity analyses and comparator scoring systems; quantitative interpretation is based on the AUC values shown on the x-axis and at the end of each bar. AUC, area under the receiver operating characteristic curve; PAD, Placental Abruption DIC; ISTH, International Society on Thrombosis and Haemostasis; DIC, disseminated intravascular coagulation.

A sensitivity analysis was also performed in the subgroup with histopathological confirmation of placental abruption at delivery. In this most stringently confirmed subgroup, the full multivariable PAD model achieved an optimism-corrected AUC of 0.887, compared with 0.916 in the primary full-cohort analysis. The discriminative performance of the model was therefore largely preserved in the subset with the most stringent diagnostic confirmation, and the distributional characteristics of placental separation percentage and the other predictors mirrored those of the full cohort. This indicates that the model performance is not driven by inclusion of cases with less stringent diagnostic confirmation. We note that this subgroup analysis (*n* = 152; 35 DIC events) provides an events-per-variable ratio of 7.0 for the five-predictor full model, which is slightly below the conventional ≥10 events-per-variable threshold; the optimism-corrected AUC of 0.887 in this subgroup should therefore be interpreted as supportive rather than confirmatory. To simultaneously address the events-per-variable, structural-overlap, and diagnostic-confirmation concerns in a single analysis, we additionally fitted the reduced three-predictor model (shock index, placental separation percentage, and chronic hypertension or preeclampsia; events-per-variable ratio = 11.7) restricted to the same pathology-confirmed subgroup. The reduced model yielded an apparent AUC of 0.896, an optimism-corrected AUC of 0.887 (95% CI 0.840–0.934; bootstrap optimism 0.009), indicating that the discriminative performance observed in the most stringently confirmed subgroup is preserved even after removal of the two ISTH-overlapping predictors. The full numerical summary of the primary, sensitivity, comparator, reduced-model, composite-outcome and pathology-subgroup analyses is provided in Supplementary Table S7 and visualized in Figure 1.

### 3.4. Comparative Discrimination Against Existing DIC Scoring Systems

The PAD score outperformed established comparator scoring systems. The AUC was 0.916 for the PAD score, 0.812 for the ISTH overt DIC score applied predictively at admission, 0.848 for the Erez pregnancy-modified DIC score, and 0.793 for the Kobayashi obstetrical DIC score [12,13,14]. Compared with the PAD score, the change in AUC was −0.104 for ISTH, −0.068 for Erez, and −0.123 for Kobayashi, with statistically significant DeLong *p* values (Supplementary Table S8). A visual summary of PAD model performance across the primary, sensitivity and comparator analyses is shown in Figure 1.

### 3.5. PAD Score Distribution and Risk-Group Performance

**Figure 2:**
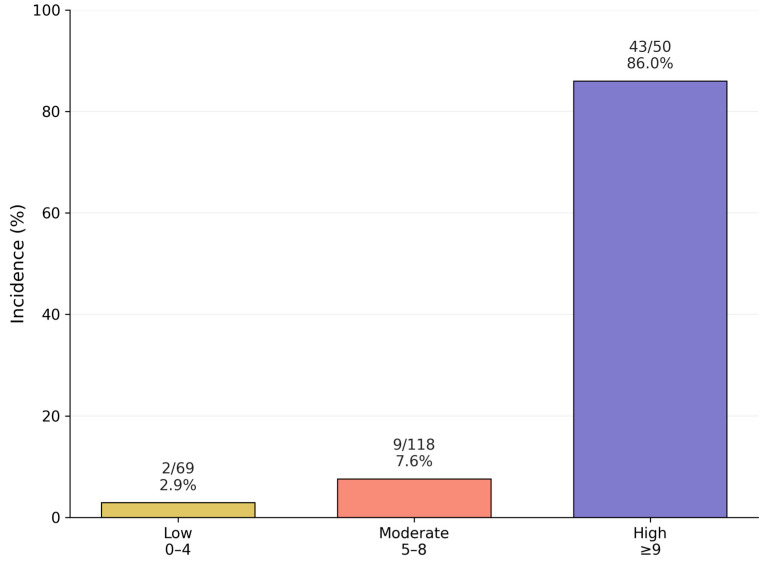
Observed overt DIC incidence stratified by PAD Score risk category. Low risk (0–4 points): 2/69 (2.9%); moderate risk (5–8 points): 9/118 (7.6%); high risk (≥9 points): 43/50 (86.0%). *p* for trend < 0.001. PAD, Placental Abruption DIC; DIC, disseminated intravascular coagulation.

DIC incidence increased sharply across risk categories: 2.9% (2/69) in the low-risk group, 7.6% (9/118) in the moderate-risk group, and 86.0% (43/50) in the high-risk group (*p* for trend < 0.001). The distribution of scores and observed DIC incidence is summarized in Table 7. The observed gradient is illustrated in Figure 2.

Diagnostic performance characteristics of three clinically meaningful PAD score cut-offs are summarized in Table 8.

**Figure 3:**
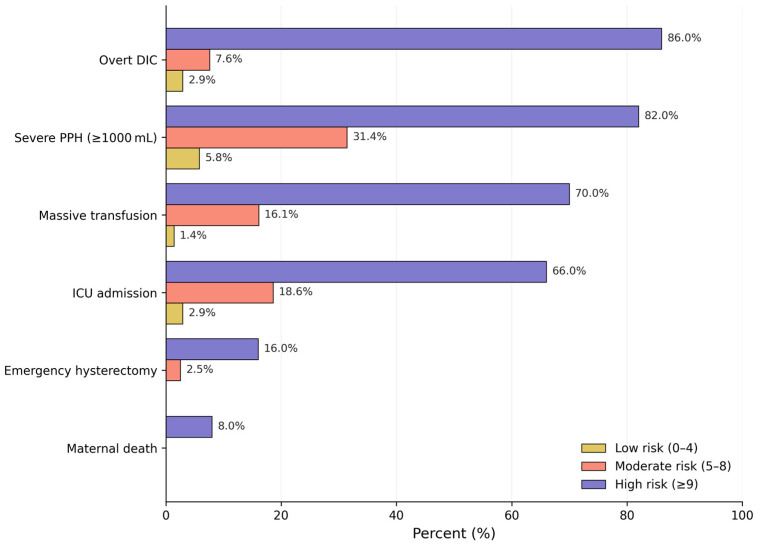
Maternal outcomes stratified by PAD score risk group. Bars represent observed proportions within each category; values correspond to those in Table 9. PAD, Placental Abruption DIC; DIC, disseminated intravascular coagulation; MTP, massive transfusion protocol; ICU, intensive care unit.

Clinically relevant maternal outcomes also increased across PAD risk groups. Severe postpartum hemorrhage occurred in 5.8% of the low-risk group, 31.4% of the moderate-risk group, and 82.0% of the high-risk group. Massive transfusion protocol activation occurred in 1.4%, 16.1%, and 70.0%, intensive care unit admission in 2.9%, 18.6%, and 66.0%, emergency peripartum hysterectomy in 0.0%, 2.5%, and 16.0%, and maternal death within 42 days in 0.0%, 0.0%, and 8.0%, respectively. These outcomes are detailed in Table 9 and illustrated in Figure 3

### 3.6. Clinical Utility by Decision Curve Analysis

**Figure 4:**
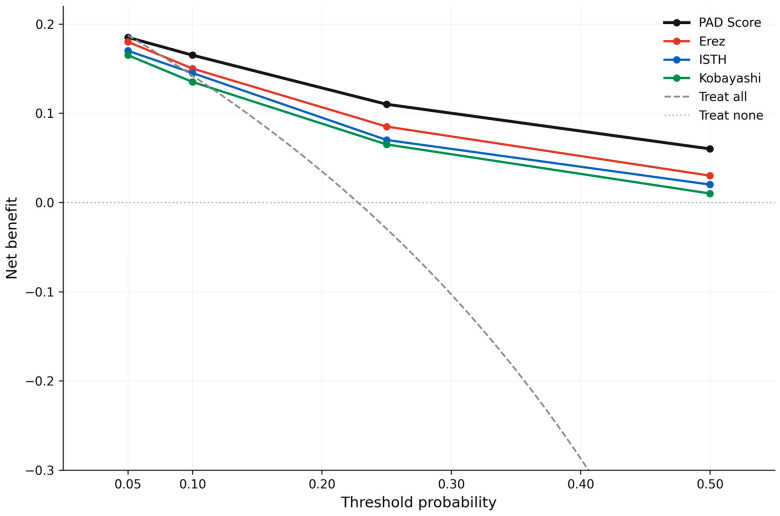
Decision curve analysis comparing the net clinical benefit of the PAD Score (solid black line) against “treat all” (gray solid line), “treat none” (light gray horizontal at zero), and three comparator scoring systems: the admission original ISTH overt DIC score (blue), the Erez pregnancy-modified DIC score (red), and the Kobayashi obstetric DIC score (green). The x-axis shows the threshold probability used for a risk-based action; the y-axis shows the net clinical benefit. The PAD Score produced the highest net benefit across the full 0.05–0.50 threshold-probability range. PAD, Placental Abruption DIC; DIC, disseminated intravascular coagulation; MTP, massive transfusion protocol; ICU, intensive care unit.

Decision curve analysis demonstrated that the PAD Score yielded a higher net clinical benefit than any comparator strategy across the full range of clinically relevant threshold probabilities examined (0.05 to 0.50) (Figure 4). At a threshold probability of 0.10—corresponding to the low-risk/moderate-risk boundary of the PAD Score—the PAD Score delivered a net benefit of 0.168, exceeding that of the Erez pregnancy-modified score (0.151), the admission ISTH overt DIC score (0.143), the Kobayashi obstetric DIC score (0.136), and the treat-all strategy (0.142). At a threshold probability of 0.25, corresponding to the moderate-risk/high-risk boundary, the PAD Score retained the highest net benefit (0.112) relative to Erez (0.083), ISTH (0.071), Kobayashi (0.064), and treat-all (−0.030). At a threshold probability of 0.50, representing an aggressive-action scenario, the PAD Score (net benefit 0.061) continued to outperform Erez (0.028), ISTH (0.019), Kobayashi (0.013), and the treat-all strategy (−0.544). The PAD Score outperformed both default strategies and all comparator scoring systems across the examined probability range, supporting its use for guiding blood-product mobilization, MTP readiness, and surgical preparedness.

## 4. Discussion

### 4.1. Summary of Principal Findings

In this retrospective cohort study of 237 women with placental abruption at a tertiary referral center in Istanbul, Turkey, we developed and internally validated the Placental Abruption DIC (PAD) Score, a clinical prediction model designed to estimate the risk of disseminated intravascular coagulation within 24 h of presentation. The model incorporates five readily available clinical, laboratory, and ultrasonographic parameters that can be assessed within minutes of patient presentation: fibrinogen concentration, shock index, platelet count, placental separation percentage, and the presence of chronic hypertension or preeclampsia. Each predictor was independently significant in multivariable analysis and biologically plausible.

The PAD Score demonstrated excellent predictive performance in internal validation. The optimism-corrected AUC was 0.916 (95% CI 0.882–0.954), indicating excellent discrimination. By standard benchmarks [41], AUC values above 0.90 represent excellent discrimination. Calibration was similarly strong, with a calibration slope of 0.96 (ideally 1.0) and intercept of −0.08 (ideally 0), indicating that predicted probabilities closely matched observed outcome frequencies across the full range of predicted risk. The Brier score of 0.12 compares favorably with other established obstetric prediction models and indicates good overall predictive accuracy [34,35].

The translation of the regression model into a simplified point-based scoring system (range 0–15 points) yielded three clinically distinct risk categories with markedly different DIC incidence rates: 2.9% in the low-risk group (0–4 points), 7.6% in the moderate-risk group (5–8 points), and 86.0% in the high-risk group (≥9 points). The gradient from 2.9% in the low-risk category to 86.0% in the high-risk category reflects the score’s clinically actionable separation of risk. The risk categories also showed consistent gradients across secondary outcomes—severe hemorrhage, massive transfusion, emergency hysterectomy, ICU admission, and maternal death—suggesting the score reflects overall disease severity rather than DIC risk alone.

The primary outcome was adjudicated using the original ISTH overt DIC scoring algorithm with the conventional cut-off of ≥5, ensuring harmonization with the broader DIC literature. Robustness of the model was further supported by a prespecified sensitivity framework in which discrimination remained stable across complete-case, single-imputation, Firth-penalized, transfer-excluded, and four-variable analyses. Comparative evaluation against the admission ISTH, Erez pregnancy-modified, and Kobayashi obstetric DIC scoring systems showed consistently higher discrimination for the PAD score.

### 4.2. Comparison with Existing Literature and Prediction Models

To our knowledge, the PAD Score represents the first clinical prediction model developed specifically for estimating DIC risk in the context of placental abruption. Placental abruption–associated DIC has distinct pathophysiology that differentiates it from DIC arising from other obstetric conditions such as amniotic fluid embolism, sepsis, or acute fatty liver of pregnancy [5,6,42].

#### 4.2.1. Comparison with ISTH DIC Scoring Systems

The distinction between diagnostic DIC scores and the PAD Score should be emphasized. In the present study, overt DIC was adjudicated using the original ISTH overt DIC scoring algorithm proposed by Taylor et al. [12], in which a cumulative score of ≥5 is considered compatible with overt DIC. This diagnostic algorithm was used solely for outcome classification and not for bedside risk prediction. By contrast, the PAD Score is a separate prediction model developed to estimate the probability of subsequent DIC among women presenting with placental abruption.

The International Society on Thrombosis and Haemostasis (ISTH) DIC scoring system remains the most widely utilized diagnostic tool for DIC in clinical practice [12]. However, this system was developed and validated primarily in general intensive care unit (ICU) populations, with sepsis and malignancy representing the predominant underlying etiologies. When applied to obstetric populations, the ISTH score demonstrates several important limitations. First, the physiological changes in pregnancy—including a 50% increase in fibrinogen concentration, elevated D-dimer levels, and mild gestational thrombocytopenia—alter the baseline coagulation profile from which pathological deviations must be distinguished [39,43,44]. Second, the rapid onset and progression of abruption-associated DIC may outpace the kinetics of standard coagulation tests, leading to delayed recognition [10].

Pregnancy-specific and obstetric-specific diagnostic systems have also been proposed. Erez et al. developed a pregnancy-adjusted DIC score to account for gestational changes in hemostasis and reported that a score of ≥26 was associated with a high probability of DIC [13]. Kobayashi proposed an obstetrical DIC score that combines clinical findings, the underlying obstetric condition, and laboratory parameters, using a different diagnostic framework in which treatment/diagnostic action is typically considered at scores of ≥8 [14,45]. To avoid conflating these distinct systems, the diagnostic score used for outcome adjudication should be clearly distinguished from the PAD Score, which is intended as a predictive bedside tool.

In direct head-to-head comparison within the present cohort, these comparator systems showed lower discrimination than the PAD score (AUC 0.812 for ISTH applied predictively at admission, 0.848 for Erez, and 0.793 for Kobayashi, versus 0.916 for PAD), with statistically significant DeLong differences. The performance gap reflects the fundamental difference between tools designed to confirm existing coagulopathy and a model built specifically for admission-based risk prediction in abruption.

The fundamental distinction between diagnostic and predictive tools is clinically important: diagnostic scores confirm the presence of a condition that has already developed, whereas prediction models identify patients at elevated risk before the condition manifests, enabling proactive intervention.

#### 4.2.2. Comparison with Obstetric Early Warning Systems

Several obstetric early warning systems have been developed to identify women at risk of clinical deterioration, including the Modified Early Obstetric Warning System (MEOWS), the Maternal Early Warning Criteria (MEWC), and various institution-specific early warning scores [46,47]. These tools have proven useful for triggering clinical review in deteriorating patients. For example, the MEWC, endorsed by the National Partnership for Maternal Safety, specifies trigger thresholds for vital signs and clinical parameters that should prompt bedside evaluation [47]. Singh et al. validated an obstetric early warning system in over 100,000 deliveries and demonstrated reduced maternal morbidity with systematic implementation [48].

However, obstetric early warning systems are designed as general screening instruments applicable to heterogeneous populations of pregnant and postpartum women. They are not disease-specific and do not include predictors particularly relevant to individual diagnoses such as placental abruption. The PAD Score addresses a more focused clinical question—the probability of DIC development in women already diagnosed with placental abruption—and incorporates disease-specific predictors such as placental separation percentage and fibrinogen concentration that would not be included in general early warning systems. This focused approach yields better predictive performance for the specific outcome in question.

#### 4.2.3. Comparison with Postpartum Hemorrhage Prediction Models

Prediction models for PPH represent the most closely analogous prior work to the PAD Score. Dilla et al. developed a risk stratification tool for severe PPH using prenatal and intrapartum variables, demonstrating that clinical risk assessment could identify women who would benefit from enhanced preparation [49]. Neary et al. conducted a systematic review of PPH prediction models, identifying several tools with moderate predictive performance but noting significant heterogeneity in outcome definitions and predictor selection [50]. More recently, Venkatesh et al. developed and validated a prediction model for severe maternal morbidity in women with PPH, incorporating variables such as body mass index, multiple gestation, and placenta previa [51].

Notably, the California Maternal Quality Care Collaborative (CMQCC) developed an obstetric hemorrhage risk assessment tool that stratifies women into low, medium, and high-risk categories based on prenatal factors [52]. This tool has been widely implemented and has contributed to reductions in severe maternal morbidity in California. However, it addresses hemorrhage broadly rather than the specific complication of DIC, and it was not designed for the placental abruption population. The pathophysiology of abruption-associated hemorrhage differs from other causes of obstetric bleeding in its propensity for consumptive coagulopathy due to massive tissue factor release from the damaged placental bed [6]. The PAD Score addresses this gap directly, offering a prediction tool tailored to the pathophysiology and management priorities of abruption.

### 4.3. Interpretation of Individual Predictors

Each of the five retained predictors is biologically plausible, supported by prior evidence, and measurable at the bedside. We emphasize that the term “non-overlapping”, as used elsewhere in this manuscript for the reduced three-predictor model, refers to the absence of shared variables with the ISTH scoring algorithm rather than to independence from the underlying pathophysiological cascade. Shock index reflects hemodynamic compromise that is itself partly driven by consumptive coagulopathy; placental separation percentage is an upstream mechanistic driver of DIC via massive tissue-factor release from disrupted decidual tissue; and chronic hypertension/preeclampsia confers baseline endothelial and hemostatic dysfunction. The optimism-corrected AUC of 0.842 obtained with these three predictors should therefore be understood as reflecting the discriminative value of admission-available clinical markers, not as a claim of biological independence from the DIC outcome.

#### Fibrinogen Concentration

Fibrinogen emerged as the strongest predictor in our model, with levels below 150 mg/dL conferring the highest point contribution (4 points) to the PAD Score. This finding is consistent with prior evidence that hypofibrinogenemia predicts hemorrhage severity and transfusion requirements in obstetric bleeding. The reasons for fibrinogen’s predictive strength in this setting are well characterized.

In their landmark prospective study, Charbit et al. evaluated 128 women with postpartum hemorrhage (PPH) and found that fibrinogen concentration at the onset of bleeding was the only laboratory parameter independently associated with progression to severe hemorrhage [40]. A fibrinogen level below 200 mg/dL had a positive predictive value of 100% for severe hemorrhage in their cohort. This finding has been consistently replicated in subsequent studies. Collins et al., in the OBS2 randomized controlled trial, demonstrated that early fibrinogen replacement guided by viscoelastometric testing improved outcomes in postpartum hemorrhage (PPH) [11]. De Lloyd et al. showed that conventional coagulation tests, including fibrinogen, performed within the first hour of major obstetric hemorrhage could predict the need for massive transfusion [53].

Normal pregnancy is characterized by progressive increases in fibrinogen concentration, reaching levels of 400–600 mg/dL by the third trimester—approximately 50% higher than non-pregnant values [43,44]. This physiological hyperfibrinogenemia provides a protective buffer against hemorrhage-induced coagulopathy and explains why fibrinogen levels that would be normal in non-pregnant adults are associated with increased bleeding risk in pregnancy. In placental abruption, the release of tissue factor-rich decidual fragments into the maternal circulation triggers explosive thrombin generation and fibrin deposition, rapidly consuming fibrinogen and other clotting factors [5,6]. The degree of fibrinogen depletion at presentation therefore reflects both the severity of the abruption and the extent to which compensatory hemostatic mechanisms have been exhausted.

Point-of-care fibrinogen testing, including viscoelastic hemostatic assays such as thromboelastography (TEG) and rotational thromboelastometry (ROTEM), can provide rapid fibrinogen assessment and may facilitate earlier application of the PAD Score in clinical practice [11,54]. The FIBTEM A5 parameter on ROTEM and the functional fibrinogen component on TEG correlate well with Clauss fibrinogen measurements and can provide results within 10–15 min rather than the 30–60 min typically required for conventional laboratory testing. Implementation of point-of-care testing may enhance the clinical utility of fibrinogen-based risk stratification in emergency settings.

### 4.4. Clinical Implications and Proposed Management Algorithm

The PAD score should be interpreted as an admission-based short-horizon risk stratification tool rather than as a replacement for the diagnosis of overt DIC already present at presentation. This design—admission predictors forecasting an outcome within 24 h—parallels the structure of established short-horizon tools such as NEWS2, qSOFA, and the CMQCC hemorrhage risk-assessment tool. The intended bedside use of the score is to trigger pro-active mobilization of blood products, surgical readiness, and intensified monitoring in women who appear stable at presentation but carry a high probability of progression to overt coagulopathy within hours.

The individual score components can also inform specific therapeutic decisions. For example, a high point contribution from fibrinogen (indicating hypofibrinogenemia) might prompt early fibrinogen replacement with cryoprecipitate or fibrinogen concentrate, potentially before the patient meets criteria for massive transfusion protocol (MTP) activation [11,55]. Proactive fibrinogen replacement has been shown to improve hemostasis and reduce transfusion requirements in obstetric hemorrhage.

Similarly, a high shock index contribution might trigger aggressive volume resuscitation, early vasopressor support, consideration of arterial line placement for continuous blood pressure monitoring, and preparation for potential surgical intervention. The identification of hemodynamic compromise at presentation allows for early optimization of the patient’s cardiovascular status before further deterioration occurs [56].

For patients with high scores driven primarily by extensive placental separation but relatively preserved laboratory values, close monitoring with serial laboratory assessment may be particularly important, as the clinical situation may evolve rapidly as thromboplastin continues to be released from the separation site. These patients may benefit from early delivery to limit ongoing exposure to procoagulant material from the abruption site.

### 4.5. Comparison with Machine Learning Approaches

Machine learning methods are increasingly applied in clinical prediction research. We chose to develop the PAD Score using logistic regression because the intended end product was a transparent, point-based bedside tool that can be applied rapidly without computational support. In emergency obstetric settings, this level of interpretability is a major practical advantage.

Recent machine learning approaches can provide clear added value in high-dimensional discovery settings. For example, Qiu et al. successfully applied a dual-algorithm consensus strategy combining XGBoost and deep neural networks to identify platelet transcriptomic biomarkers across inflammatory conditions [57]. However, that context differs fundamentally from bedside DIC prediction in placental abruption, where the feature space is intentionally low-dimensional and the intended output must remain immediately interpretable.

Our study included 237 patients with 54 outcome events, and the continuous predictors showed no important departures from linearity. Under these conditions, more complex machine learning models would be at substantial risk of overfitting and would be unlikely to yield stable advantages over logistic regression [58,59].

In addition, the clinical acceptance and implementation of prediction models depend partly on clinicians’ ability to understand and trust the model’s recommendations. The transparency of logistic regression—where clinicians can see which factors contribute to predicted risk and in what direction—may facilitate adoption in a way that opaque models cannot [60,61].

We acknowledge that future studies with larger, multicenter datasets may identify useful non-linear effects or interactions and may profitably explore machine learning methods. However, for the current purpose of deriving a practical score from a moderately sized single-center cohort, logistic regression remained the most appropriate methodological choice.

### 4.6. Strengths of the Study

Several methodological features merit acknowledgment. First, the study was conducted and reported in adherence to the TRIPOD guidelines, with a completed checklist provided as Supplementary Table S1 [20,21].

Second, we employed rigorous internal validation using bootstrap resampling with 1000 iterations, which provides a nearly unbiased estimate of model performance in new patients and quantifies the degree of optimism (overfitting) in the apparent performance metrics [32,33]. The minimal optimism observed (0.008 for AUC) suggests that the model is unlikely to perform substantially worse when applied to new patients from similar clinical settings. Bootstrap validation is recommended over split-sample validation for moderate sample sizes because it uses all available data for both model development and validation, providing more stable estimates.

Third, we evaluated model performance using multiple complementary metrics, including discrimination (AUC), calibration (calibration plot, slope, intercept, Brier score), and clinical utility (decision curve analysis). Reliance on discrimination alone has been criticized as insufficient for evaluating prediction models intended for clinical use [34,35]; this multidimensional assessment provides a more complete performance picture. A model with excellent discrimination but poor calibration may misclassify patients’ absolute risk, potentially leading to inappropriate clinical decisions.

Fourth, all candidate predictors were restricted to variables available at the time of initial evaluation, enabling early risk stratification when clinical decisions are most consequential. The inclusion of placental separation percentage, while requiring ultrasonographic expertise, is appropriate given that ultrasound is a standard component of the evaluation of suspected placental abruption at most obstetric facilities.

Fifth, the outcome definition (overt DIC within 24 h of admission) was based on objective laboratory criteria using the original ISTH overt DIC scoring algorithm, minimizing ascertainment bias that could arise from subjective outcome assessment. The temporal separation between predictor assessment (admission values) and outcome determination (worst values after admission) ensures appropriate directionality of the predictive relationship.

Sixth, we assessed inter-observer reliability for key predictor measurements, including placental separation percentage, and demonstrated excellent agreement (ICC 0.91) between independent observers. This provides confidence that the predictor measurements can be reproduced reliably in clinical practice.

### 4.7. Limitations of the Study

Several limitations should be considered when interpreting these findings. First, the single-center design limits the external validity of our findings. Our institution is a tertiary referral center with specialized perinatology services, 24 h availability of maternal-fetal medicine subspecialists, and a comprehensive blood bank. The patient population—including a high proportion of transferred patients (37.6%) and those with severe disease—may not be representative of placental abruption cases managed at community hospitals or primary care facilities. The PAD Score’s performance may differ in settings with different patient demographics, referral patterns, clinical practices, or resource availability.

Second, the retrospective design introduces potential for information bias, as predictor variables and outcomes were extracted from existing medical records rather than prospectively collected using standardized protocols. Although we used trained research personnel and standardized data extraction forms with demonstrated inter-rater reliability, some degree of measurement error is inevitable with retrospective data collection. In particular, the timing of laboratory specimen collection relative to blood product transfusion could not always be precisely determined, potentially affecting the accuracy of admission coagulation parameters in some cases.

Third, the sample size, while adequate for development of a five-predictor model by conventional events-per-variable criteria, is relatively modest and limits our ability to detect smaller effect sizes, evaluate potential interactions between predictors, or develop more complex models that might capture additional prognostic information. The confidence intervals around our performance estimates are correspondingly wide, and the true performance of the model in new patients could fall anywhere within these intervals.

Fourth, and most critically, the PAD Score has not undergone external validation in independent cohorts, which is essential before recommending clinical implementation. Internal validation methods such as bootstrap resampling provide optimism-corrected performance estimates but cannot fully substitute for external validation in geographically or temporally distinct populations [20,28]. The performance of prediction models typically degrades when applied to new populations, a phenomenon known as ‘transportability’ or ‘generalizability’ failure, and the magnitude of this degradation cannot be estimated without external validation data.

Fifth, the reliance on ultrasonographic estimation of placental separation percentage introduces potential measurement variability that could affect score reproducibility. Although we achieved excellent inter-observer reliability in our study through standardized protocols and experienced sonographers, this level of reliability may be difficult to replicate in routine clinical practice, particularly in emergency settings or facilities with less specialized ultrasound expertise.

Sixth, we did not evaluate the potential added value of emerging biomarkers such as viscoelastic testing parameters (TEG/ROTEM), prothrombin fragment 1+2, thrombin-antithrombin complexes, or soluble fibrin monomers, which might provide earlier indication of evolving DIC than conventional coagulation tests [53,62]. The inclusion of such markers in future iterations of the PAD Score could potentially improve predictive performance.

Seventh, although the primary outcome definition was harmonized using the original ISTH overt DIC algorithm, two of the retained predictors—fibrinogen concentration and platelet count—are themselves components of that algorithm, creating a structural overlap between predictors and outcome that has the potential to inflate apparent discrimination. We addressed this concern empirically rather than rhetorically. A reduced multivariable model that retained only the three non-overlapping predictors (shock index, placental separation percentage, and chronic hypertension or preeclampsia) achieved an optimism-corrected AUC of 0.842 (ΔAUC versus the full model −0.074, DeLong *p* < 0.001), and the full PAD Score discriminated an independent composite clinical outcome (massive transfusion, hysterectomy, prolonged ICU admission, or maternal death) with an AUC of 0.801. The reduced-model AUC of 0.842 is itself in the range conventionally regarded as good-to-excellent discrimination and exceeds that of the established comparator scoring systems applied predictively at admission, indicating that the non-overlapping predictors carry substantial independent prognostic information. We nonetheless interpret the full-model performance with appropriate caution: a portion of the apparent discrimination of the full PAD Score is, by construction, attributable to the structural overlap between platelet count, fibrinogen, and the ISTH outcome adjudication, and external validation in independent cohorts is essential before this overlap is presumed to be without clinical consequence.

Eighth, placental abruption is diagnosed in routine tertiary emergency practice using clinical, ultrasonographic, and, when available, pathological criteria rather than a single reference standard, and pathological confirmation was not available for every case. The full breakdown of confirmation modalities in our cohort is reported in Section Diagnostic Confirmation of Placental Abruption and Distribution of Placental Separation and Supplementary Table S2, and a sensitivity analysis restricted to the histopathology-confirmed subgroup (Section 3.3) yielded an optimism-corrected AUC of 0.887, indicating that the discriminative performance of the model is largely preserved under the most stringent diagnostic standard. The distribution of placental separation percentage across the cohort is provided as Supplementary Figure S2. Some degree of diagnostic heterogeneity is nevertheless unavoidable in real-world cohorts such as ours, and the PAD score should be interpreted with this context in mind.

### 4.8. Future Research Directions

Several directions for future research emerge from this work. The most critical and immediate next step is external validation of the PAD Score in independent cohorts from diverse clinical settings. Ideally, this would include prospective validation studies at multiple centers representing different levels of care (community hospitals, regional referral centers, and academic medical centers), different geographic regions, and different patient populations. Multicenter validation studies would provide more robust estimates of the score’s transportability and identify any systematic differences in performance across populations or practice settings that might require model recalibration [63].

If external validation confirms adequate performance, implementation studies would be valuable to assess the real-world impact of PAD Score-guided management on clinical outcomes. Such studies might employ stepped-wedge cluster randomized designs or interrupted time series analyses to evaluate whether systematic application of the PAD Score improves time to treatment initiation, reduces DIC-related morbidity, optimizes blood product utilization, or improves maternal outcomes compared with usual care [64].

Integration of the PAD Score into electronic health record systems could facilitate automatic calculation and display of risk scores at the point of care, potentially with clinical decision support alerts for high-risk patients. Human factors research would be valuable to optimize the design of such alerts and ensure that they effectively support clinical decision-making without contributing to alert fatigue, which has been identified as a significant barrier to clinical decision support effectiveness [65].

The potential role of additional biomarkers in improving PAD Score performance warrants investigation. Emerging markers of coagulation activation, such as prothrombin fragment 1+2, thrombin-antithrombin complexes, and soluble fibrin monomers, might provide earlier indication of evolving DIC than conventional coagulation tests [62]. Similarly, point-of-care viscoelastic testing (TEG/ROTEM) parameters could potentially be incorporated into future iterations of the score to enable more rapid risk stratification in settings where this technology is available [53].

Finally, economic evaluation of PAD Score-guided management would help inform implementation decisions and resource allocation. Cost-effectiveness analysis should consider the costs of score implementation (training, potential delays in care, false-positive interventions) against the potential benefits of earlier intervention for true-positive cases (reduced transfusion requirements, fewer hysterectomies, shorter ICU stays, lives saved). Such analyses would be particularly valuable for resource-limited settings where the efficient allocation of scarce resources is paramount [66].

## 5. Conclusions

Placental abruption remains among the most serious obstetric emergencies, and DIC is among its most feared complications—one that worsens outcomes substantially when recognized late. In this study, we developed and internally validated the Placental Abruption DIC (PAD) Score, a clinical prediction model designed to estimate the risk of DIC development in women presenting with placental abruption. The score fills a practical gap: an objective, quantitative tool for stratifying DIC risk at the time of admission, before coagulopathy becomes apparent.

The PAD Score incorporates five readily available parameters that can be assessed within minutes of patient presentation: fibrinogen concentration, shock index, platelet count, ultrasonographically estimated placental separation percentage, and the presence of chronic hypertension or preeclampsia. Each is biologically plausible and supported by prior evidence. The simplified 0–15 point system enables rapid bedside calculation without electronic decision support, which is practical in emergency settings.

The model demonstrated excellent predictive performance in internal validation, with an optimism-corrected area under the ROC curve (AUC) of 0.916, calibration slope of 0.96, and Brier score of 0.12. Decision curve analysis confirmed clinical utility across a wide range of threshold probabilities (Figure 4). The three risk categories derived from the PAD Score—low (0–4 points), moderate (5–8 points), and high (≥9 points)—demonstrated markedly different DIC incidence rates of 2.9%, 7.6%, and 86.0%, respectively. These risk strata also showed strong associations with secondary maternal outcomes including severe hemorrhage, massive transfusion requirement, emergency hysterectomy, ICU admission, and maternal mortality.

We acknowledge important limitations, including single-center design, retrospective data collection, and the absence of external validation. External validation in independent populations is essential before the PAD Score can be recommended for routine clinical use. Until such validation is available, the score should be viewed as a research tool and a framework for standardizing risk assessment rather than a validated clinical instrument.

The structural overlap between two of the predictors (fibrinogen, platelet count) and the ISTH outcome adjudication was addressed empirically through a reduced three-predictor model that retained good-to-excellent discrimination (AUC 0.842) and through evaluation of the full PAD Score against an independent composite clinical outcome (AUC 0.801), demonstrating that the score captures prognostic information that is not fully reducible to its overlapping laboratory components. Diagnostic-confirmation heterogeneity was addressed through a histopathology-confirmed subgroup analysis (AUC 0.887). These analyses do not eliminate the underlying methodological tensions, but they provide transparent quantitative evidence that the score performs robustly across alternative analytic specifications.

The PAD Score offers a practical, evidence-based approach to early DIC risk stratification in placental abruption. The score integrates five bedside parameters into a simple point-based system that demonstrated excellent discrimination and calibration in internal validation. High-risk classification identified women at markedly elevated risk of DIC, severe hemorrhage, and maternal mortality. External validation in independent cohorts is the essential next step before clinical implementation. We hope these findings support earlier identification of high-risk women and more timely, targeted clinical responses.

## Figures and Tables

**Table 1:** Original ISTH Overt DIC Scoring Algorithm Used for Outcome Adjudication.

Component	Category	Points
Platelet count	>100 × 10^9^/L	0
Platelet count	<100 × 10^9^/L	1
Platelet count	<50 × 10^9^/L	2
Fibrin-related markers *	No increase	0
Fibrin-related markers *	Moderate increase	2
Fibrin-related markers *	Strong increase	3
Prolonged prothrombin time	<3 s	0
Prolonged prothrombin time	3–6 s	1
Prolonged prothrombin time	>6 s	2
Fibrinogen level	>1.0 g/L	0
Fibrinogen level	<1.0 g/L	1

* Fibrin-related markers refer to fibrin degradation products or D-dimer, according to the original ISTH overt DIC scoring algorithm [12]. ISTH = International Society on Thrombosis and Haemostasis. DIC = disseminated intravascular coagulation. Interpretation: Total score ≥ 5: compatible with overt DIC; total score < 5: suggestive, but not diagnostic, of non-overt DIC.

**Table 2:** Baseline demographic and clinical characteristics of women with placental abruption at admission, stratified by subsequent development of overt disseminated intravascular coagulation within 24 h.

Characteristic	No DIC (*n* = 183)	DIC (*n* = 54)	SMD	Test	*p*-Value
Maternal age, years, mean ± SD	29.6 ± 5.5	30.2 ± 5.8	0.11	*t*-test	0.48
Body mass index, kg/m^2^, mean ± SD	27.1 ± 4.2	27.8 ± 4.5	0.16	*t*-test	0.33
Nulliparous, *n* (%)	77 (42.1)	21 (38.9)	0.07	χ^2^	0.67
Smoking during pregnancy, *n* (%)	27 (14.8)	9 (16.7)	0.05	χ^2^	0.74
Diabetes (pregestational or gestational), *n* (%)	22 (12.0)	7 (13.0)	0.03	χ^2^	0.85
Chronic hypertension/preeclampsia, *n* (%)	44 (24.0)	23 (42.6)	0.40	χ^2^	0.008
Prior placental abruption, *n* (%)	10 (5.5)	8 (14.8)	0.31	Fisher	0.027
Transfer from external facility, *n* (%)	61 (33.3)	28 (51.9)	0.38	χ^2^	0.014
Gestational age at presentation, wk, mean ± SD	35.2 ± 3.8	32.5 ± 4.2	0.67	*t*-test	0.003
Hemoglobin at admission, g/dL, mean ± SD	10.8 ± 1.8	8.4 ± 2.3	1.16	*t*-test	<0.001
Platelet count, ×10^9^/L, median (IQR)	198 (156–242)	89 (62–134)	1.86	Mann–Whitney	<0.001
Fibrinogen, mg/dL, median (IQR)	324 (278–371)	142 (98–186)	2.72	Mann–Whitney	<0.001
Fibrinogen < 200 mg/dL, *n* (%)	12 (6.6)	38 (70.4)	1.74	χ^2^	<0.001
Shock index, mean ± SD	0.78 ± 0.12	1.02 ± 0.25	1.22	*t*-test	<0.001
Shock index ≥ 1.0, *n* (%)	16 (8.7)	22 (40.7)	0.80	χ^2^	<0.001
Placental separation, %, mean ± SD	21 ± 14	42 ± 18	1.30	*t*-test	<0.001

DIC, disseminated intravascular coagulation; IQR, interquartile range; SD, standard deviation; SMD, standardized mean difference.

**Table 3:** Univariable screening of candidate predictors for overt disseminated intravascular coagulation within 24 h.

Candidate Predictor (Increment)	Unadjusted OR	95% CI	Wald *p*	Retained
Fibrinogen (per 10 mg/dL decrease)	0.984	0.979–0.989	<0.001	Yes
Shock index (per 0.1 unit increase)	6.51	4.24–10.00	<0.001	Yes
Platelet count (per 10 ×10^9^/L decrease)	0.975	0.968–0.982	<0.001	Yes
Placental separation (per 10% increase)	1.55	1.35–1.79	<0.001	Yes
Chronic hypertension/preeclampsia	2.34	1.27–4.33	0.006	Yes
Prior placental abruption	2.99	1.13–7.91	0.027	No
Transfer from external facility	2.16	1.17–3.98	0.014	No
Gestational age (per 1 week decrease)	1.19	1.08–1.31	<0.001	No
Hemoglobin at admission (per 1 g/dL decrease)	1.79	1.48–2.17	<0.001	No
D-dimer (per 1 µg/mL increase)	1.12	1.06–1.18	<0.001	No
Prothrombin time (per 1 s increase)	1.28	1.17–1.40	<0.001	No
Activated partial thromboplastin time (per 1 s)	1.09	1.03–1.16	0.003	No
International normalized ratio (per 0.1 unit)	1.22	1.13–1.32	<0.001	No
Maternal age (per 5 years increase)	1.08	0.81–1.44	0.59	No
Nulliparity	0.88	0.48–1.64	0.69	No
Body mass index (per 1 kg/m^2^ increase)	1.03	0.96–1.11	0.40	No
Diabetes (pregestational or gestational)	1.10	0.44–2.74	0.84	No

OR, odds ratio; CI, confidence interval.

**Table 4:** Multivariable logistic regression model for prediction of overt disseminated intravascular coagulation within 24 h.

Predictor (Increment)	β	SE	Wald χ^2^	OR	95% CI	*p*-Value	VIF
Fibrinogen (per 10 mg/dL decrease)	−0.013	0.002	34.3	0.987	0.982–0.991	<0.001	1.42
Shock index (per 0.1 unit increase)	1.573	0.206	58.3	4.82	3.21–7.24	<0.001	1.18
Platelet count (per 10 × 10^9^/L decrease)	−0.019	0.004	24.1	0.981	0.974–0.988	<0.001	1.51
Placental separation (per 10% increase)	0.324	0.078	17.3	1.38	1.19–1.61	<0.001	1.09
Chronic hypertension/preeclampsia	0.850	0.254	11.2	2.34	1.42–3.86	0.001	1.24

Model intercept: −3.842. Hosmer–Lemeshow *p* = 0.55. Abbreviations: OR, odds ratio; CI, confidence interval; VIF, variance inflation factor; β, regression coefficient; SE, standard error; Wald χ^2^, Wald chi-square statistic.

**Table 5:** Internal validation and model performance of the primary PAD prediction model.

Performance Metric	Apparent Estimate	Optimism-Corrected Estimate (95% CI)
C-statistic (AUC)	0.924 (0.889–0.959)	0.916 (0.882–0.954)
Calibration intercept	0.000	−0.08
Calibration slope	1.00	0.96 (0.81–1.12)
Brier score	0.11	0.12
Nagelkerke R^2^	0.65	—
Somers’ D	0.848	0.832
Hosmer–Lemeshow χ^2^ (df = 8)	6.92 (*p* = 0.55)	—
Bootstrap shrinkage factor	—	0.94

AUC, area under the receiver operating characteristic curve; CI, confidence interval; df, degrees of freedom.

**Table 6:** Integer point assignment used to construct the PAD score.

Predictor	Category	Category Midpoint	β × (Midpoint − Reference)	Points
Fibrinogen	≥250 mg/dL	275	0.000	0
	200–249 mg/dL	225	0.065	1
	150–199 mg/dL	175	0.130	2
	<150 mg/dL	125	0.195	4
Shock index	<0.70	0.65	0.000	0
	0.70–0.89	0.80	2.36	1
	0.90–0.99	0.95	4.72	2
	≥1.00	1.15	7.87	3
Platelet count	≥200 × 10^9^/L	225	0.000	0
	150–199 × 10^9^/L	175	0.095	1
	100–149 × 10^9^/L	125	0.190	2
	<100 × 10^9^/L	75	0.285	3
Placental separation	<10%	5	0.000	0
	10–19%	15	0.324	1
	20–39%	30	0.810	2
	≥40%	55	1.620	3
Chronic hypertension/preeclampsia	Absent	—	0.000	0
	Present	—	0.850	2
Total score range	—	—	—	0–15

**Table 7:** Distribution of PAD score and observed risk of overt disseminated intravascular coagulation.

Risk Category	Score Range	*n*	DIC Events, *n*	DIC Incidence, % (95% CI)
Low	0–4	69	2	2.9 (0.4–10.1)
Moderate	5–8	118	9	7.6 (3.5–13.9)
High	≥9	50	43	86.0 (73.3–94.2)
Cochran–Armitage trend test	—	—	—	*p* < 0.001

PAD, Placental Abruption DIC; DIC, disseminated intravascular coagulation; CI, confidence interval.

**Table 8:** Diagnostic performance of clinically relevant PAD score thresholds.

Cut-Off (Score≥)	Sensitivity %	Specificity %	PPV %	NPV %	LR+	LR−
≥5	96.3 (87.3–99.5)	36.6 (29.7–44.0)	31.0 (24.1–38.6)	97.1 (90.1–99.7)	1.52 (1.34–1.72)	0.10 (0.03–0.40)
≥7 (Youden)	88.9 (77.4–95.8)	78.1 (71.5–83.9)	54.5 (44.6–64.2)	95.9 (91.3–98.5)	4.06 (3.06–5.38)	0.14 (0.07–0.30)
≥9	79.6 (66.5–89.4)	96.2 (92.2–98.4)	86.0 (73.3–94.2)	94.1 (89.6–96.9)	20.88 (9.94–43.88)	0.21 (0.13–0.35)

PAD, Placental Abruption; PPV, positive predictive value, NPV, negative predictive value; LR+, positive likelihood ratio; LR−, negative likelihood ratio.

**Table 9:** Maternal outcomes stratified by PAD risk group.

Maternal Outcome	Low, 0–4 (*n* = 69)	Moderate, 5–8 (*n* = 118)	High, ≥9 (*n* = 50)	*p* Trend
Overt disseminated intravascular coagulation	2 (2.9, 0.4–10.1)	9 (7.6, 3.5–13.9)	43 (86.0, 73.3–94.2)	<0.001
Severe postpartum hemorrhage (≥1000 mL)	4 (5.8, 1.6–14.2)	37 (31.4, 23.1–40.5)	41 (82.0, 68.6–91.4)	<0.001
Massive transfusion protocol activation	1 (1.4, 0.0–7.8)	19 (16.1, 10.0–24.0)	35 (70.0, 55.4–82.1)	<0.001
Emergency peripartum hysterectomy	0 (0.0, 0.0–5.2)	3 (2.5, 0.5–7.3)	8 (16.0, 7.2–29.1)	<0.001
Intensive care unit admission	2 (2.9, 0.4–10.1)	22 (18.6, 12.0–26.9)	33 (66.0, 51.2–78.8)	<0.001
Maternal death within 42 days	0 (0.0, 0.0–5.2)	0 (0.0, 0.0–3.1)	4 (8.0, 2.2–19.2)	<0.001

Values in parentheses are % (95% CI). PAD, Placental Abruption DIC; DIC, disseminated intravascular coagulation; CI, confidence interval.

## Data Availability

The data presented in this study are available from the corresponding author upon reasonable request. The data are not publicly available due to privacy and ethical restrictions.

## Supplementary Materials

The following supporting information can be downloaded at: https://www.mdpi.com/article/10.3390/jcm15093524/s1, including Table S1: Completed TRIPOD 22-item checklist; Table S2: Diagnostic confirmation modalities of placental abruption (ultrasonographic only, histopathological only, both) and categorical distribution of placental separation percentage, stratified by DIC status; Table S3: Robustness of overt DIC outcome classification across the original ISTH (≥5 and ≥6), Erez and Kobayashi scoring systems with Cohen’s κ agreement statistics; Table S4: Likelihood-ratio tests for nonlinearity of continuous predictors under restricted cubic splines with 3, 4, and 5 knots; Table S5: Missing-data pattern and imputation strategy; Table S6: Distribution of the original ISTH overt DIC score (0–11) stratified by final DIC classification; Table S7: Sensitivity analyses for discrimination and calibration (primary, complete-case, single imputation, Firth-penalized, transfer-excluded, four-variable, alternative thresholds, reduced model excluding overlapping predictors, composite clinical outcome, and pathology-confirmed subgroup); and Table S8: Comparative discrimination against established obstetric DIC scoring systems (ISTH, Erez, Kobayashi) with DeLong, IDI and continuous NRI statistics; and Figure S1: Smooth calibration plot of the full PAD prediction model, with calibration slope, intercept, and Brier score annotated and Figure S2: Distribution of placental separation percentage in the analytic cohort, overlaid by DIC status.

## References

[1] Oyelese Y., Ananth C.V. (2006). Placental abruption. Obstet. Gynecol..

[2] Tikkanen M. (2011). Placental abruption: Epidemiology, risk factors and consequences. Acta Obstet. Gynecol. Scand..

[3] Ananth C.V., Keyes K.M., Hamilton A., Gissler M., Wu C., Liu S., Luque-Fernandez M.A., Skjærven R., Williams M.A., Tikkanen M. (2015). An international contrast of rates of placental abruption: An age-period-cohort analysis. PLoS ONE.

[4] Downes K.L., Grantz K.L., Shenassa E.D. (2017). Maternal, labor, delivery, and perinatal outcomes associated with placental abruption: A systematic review. Am. J. Perinatol..

[5] Erez O., Othman M., Rabinovich A., Leron E., Gotsch F., Thachil J. (2022). DIC in Pregnancy—Pathophysiology, Clinical Characteristics, Diagnostic Scores, and Treatments. J. Blood Med..

[6] Bick R.L. (2003). Disseminated intravascular coagulation current concepts of etiology, pathophysiology, diagnosis, and treatment. Hematol. Oncol. Clin..

[7] Rattray D.D., O’Connell C.M., Baskett T.F. (2012). Acute disseminated intravascular coagulation in obstetrics: A tertiary centre population review (1980 to 2009). J. Obstet. Gynaecol. Can..

[8] Erez O., Mastrolia S.A., Thachil J. (2015). Disseminated intravascular coagulation in pregnancy: Insights in pathophysiology, diagnosis and management. Am. J. Obstet. Gynecol..

[9] Cunningham F.G., Nelson D.B. (2015). Disseminated intravascular coagulation syndromes in obstetrics. Obstet. Gynecol..

[10] Thachil J., Toh C.H. (2009). Disseminated intravascular coagulation in obstetric disorders and its acute haematological management. Blood Rev..

[11] Collins P.W., Cannings-John R., Bruynseels D., Mallaiah S., Dick J., Elton C., Weeks A.D., Sanders J., Aawar N., Townson J. (2017). Viscoelastometric-guided early fibrinogen concentrate replacement during postpartum haemorrhage: OBS2, a double-blind randomized controlled trial. Br. J. Anaesth..

[12] Taylor F.B., Toh C.H., Hoots W.K., Wada H., Levi M. (2001). Towards definition, clinical and laboratory criteria, and a scoring system for disseminated intravascular coagulation. Thromb. Haemost..

[13] Erez O., Novack L., Beer-Weisel R., Dukler D., Press F., Zlotnik A., Than N.G., Tomer A., Mazor M. (2014). DIC score in pregnant women—A population based modification of the International Society on Thrombosis and Hemostasis score. PLoS ONE.

[14] Kobayashi T. (2014). Obstetrical disseminated intravascular coagulation score. J. Obstet. Gynaecol. Res..

[15] Levi M., Scully M. (2018). How I treat disseminated intravascular coagulation. Blood.

[16] Pacheco L.D., Saade G.R., Costantine M.M., Clark S.L., Hankins G.D.V. (2013). The role of massive transfusion protocol (MTP)s in obstetrics. Am. J. Perinatol..

[17] Royston P., Moons K.G., Altman D.G., Vergouwe Y. (2009). Prognosis and prognostic research: Developing a prognostic model. BMJ.

[18] Merriam A.A., Wright J.D., Siddiq Z., D’Alton M.E., Friedman A.M., Ananth C.V., Bateman B.T. (2018). Risk for postpartum hemorrhage, transfusion, and hemorrhage-related morbidity at low, moderate, and high volume hospitals. J. Matern.-Fetal Neonatal Med..

[19] Main E.K., Goffman D., Scavone B.M., Low L.K., Bingham D., Fontaine P.L., Gorlin J.B., Lagrew D.C., Levy B.S. (2015). National partnership for maternal safety: Consensus bundle on obstetric hemorrhage. Obstet. Gynecol..

[20] Collins G.S., Reitsma J.B., Altman D.G., Moons K.G. (2015). Transparent Reporting of a multivariable prediction model for Individual Prognosis Or Diagnosis (TRIPOD): The TRIPOD statement. BMJ.

[21] Moons K.G., Altman D.G., Reitsma J.B., Ioannidis J.P., Macaskill P., Steyerberg E.W., Vickers A.J., Ransohoff D.F., Collins G.S. (2015). Transparent Reporting of a multivariable prediction model for Individual Prognosis or Diagnosis (TRIPOD): Explanation and elaboration. Ann. Intern. Med..

[22] Peduzzi P., Concato J., Kemper E., Holford T.R., Feinstein A.R. (1996). A simulation study of the number of events per variable in logistic regression analysis. J. Clin. Epidemiol..

[23] Vittinghoff E., McCulloch C.E. (2007). Relaxing the rule of ten events per variable in logistic and Cox regression. Am. J. Epidemiol..

[24] Riley R.D., Ensor J., Snell K.I.E., Harrell F.E., Martin G.P., Reitsma J.B., Moons K.G., Collins G., Van Smeden M. (2020). Calculating the sample size required for developing a clinical prediction model. BMJ.

[25] van Smeden M., de Groot J.A., Moons K.G., Collins G.S., Altman D.G., Eijkemans M.J., Reitsma J.B. (2016). No rationale for 1 variable per 10 events criterion for binary logistic regression analysis. BMC Med. Res. Methodol..

[26] Nathan H.L., El Ayadi A., Hezelgrave N.L., Seed P., Butrick E., Miller S., Briley A., Bewley S., Shennan A.H. (2015). Shock index: An effective predictor of outcome in postpartum haemorrhage?. BJOG Int. J. Obstet. Gynaecol..

[27] Le Bas A., Chandraharan E., Addei A., Arulkumaran S. (2014). Use of the obstetric shock index as an adjunct in identifying significant blood loss in patients with massive postpartum hemorrhage. Int. J. Gynaecol. Obstet..

[28] El Ayadi A.M., Nathan H.L., Seed P.T., Butrick E.A., Hezelgrave N.L., Shennan A.H., Miller S. (2016). Vital sign prediction of adverse maternal outcomes in women with hypovolemic shock: The role of shock index. PLoS ONE.

[29] Little R.J.A. (1988). A test of missing completely at random for multivariate data with missing values. J. Am. Stat. Assoc..

[30] van Buuren S. (2018). Flexible Imputation of Missing Data.

[31] White I.R., Royston P., Wood A.M. (2011). Multiple imputation using chained equations: Issues and guidance for practice. Stat. Med..

[32] Steyerberg E.W. (2019). Clinical Prediction Models: A Practical Approach to Development, Validation, and Updating.

[33] Harrell F.E., Lee K.L., Mark D.B. (1996). Multivariable prognostic models: Issues in developing models, evaluating assumptions and adequacy, and measuring and reducing errors. Stat. Med..

[34] Harrell F.E. (2015). Regression Modeling Strategies: With Applications to Linear Models, Logistic and Ordinal Regression, and Survival Analysis.

[35] Steyerberg E.W., Vickers A.J., Cook N.R., Gerds T., Gonen M., Obuchowski N., Pencina M.J., Kattan M.W. (2010). Assessing the performance of prediction models: A framework for some traditional and novel measures. Epidemiology.

[36] Van Calster B., McLernon D.J., van Smeden M., Wynants L., Steyerberg E.W. (2019). Calibration: The Achilles heel of predictive analytics. BMC Med..

[37] Vickers A.J., Elkin E.B. (2006). Decision curve analysis: A novel method for evaluating prediction models. Med. Decis. Mak..

[38] Vickers A.J., Van Calster B., Steyerberg E.W. (2016). Net benefit approaches to the evaluation of prediction models, molecular markers, and diagnostic tests. BMJ.

[39] Sullivan L.M., Massaro J.M., D’Agostino R.B. (2004). Presentation of multivariate data for clinical use: The Framingham Study risk score functions. Stat. Med..

[40] Charbit B., Mandelbrot L., Samain E., Baron G., Haddaoui B., Keita H., Sibony O., Mahieu-Caputo D., Hurtaud-Roux M.F., Huisse M.G. (2007). The decrease of fibrinogen is an early predictor of the severity of postpartum hemorrhage. J. Thromb. Haemost..

[41] Hosmer D.W., Lemeshow S., Sturdivant R.X. (2013). Applied Logistic Regression.

[42] Clark S.L. (2014). Amniotic fluid embolism. Obstet. Gynecol..

[43] Thornton P., Douglas J. (2010). Coagulation in pregnancy. Best Pract. Res. Clin. Obstet. Gynaecol..

[44] Brenner B. (2004). Haemostatic changes in pregnancy. Thromb. Res..

[45] Asakura H., Takahashi H., Uchiyama T., Eguchi Y., Okamoto K., Kawasugi K., Madoiwa S., Wada H., DIC Subcommittee of the Japanese Society on Thrombosis and Hemostasis (2016). Proposal for new diagnostic criteria for DIC from the Japanese Society on Thrombosis and Hemostasis. Thromb. J..

[46] Carle C., Alexander P., Columb M., Johal J. (2013). Design and internal validation of an obstetric early warning score. Anaesthesia.

[47] Mhyre J.M., D’Oria R., Hameed A.B., Lappen J.R., Holley S.L., Hunter S.K., Jones R.L., King J.C., D’Alton M.E. (2014). The maternal early warning criteria: A proposal from the national partnership for maternal safety. J. Obstet. Gynecol. Neonatal Nurs..

[48] Singh S., McGlennan A., England A., Simons R. (2012). A validation study of the CEMACH recommended modified early obstetric warning system (MEOWS). Anaesthesia.

[49] Dilla A.J., Waters J.H., Yazer M.H. (2013). Clinical validation of risk stratification criteria for peripartum hemorrhage. Obstet. Gynecol..

[50] Neary C., Naheed S., McLernon D., Black M. (2021). Predicting risk of postpartum haemorrhage: A systematic review. BJOG Int. J. Obstet. Gynaecol..

[51] Venkatesh K.K., Strauss R.A., Grotegut C.A., Heine R.P., Chescheir N.C., Stringer J.S.A., Stamilio D.M., Menard K.M., Jelovsek J.E. (2020). Machine Learning and Statistical Models to Predict Postpartum Hemorrhage. Obstet. Gynecol..

[52] Main E.K., Cape V., Abreo A., Vasher J., Woods A., Carpenter A., Gould J.B. (2017). Reduction of severe maternal morbidity from hemorrhage using a state perinatal quality collaborative. Am. J. Obstet. Gynecol..

[53] De Lloyd L., Bovington R., Kaye A., Collis R.E., Rayment R., Sanders J., Rees A., Collins P.W. (2011). Standard haemostatic tests following major obstetric haemorrhage. Int. J. Obstet. Anesth..

[54] Wikkelsø A., Wetterslev J., Møller A.M., Afshari A. (2016). Thromboelastography (TEG) or thromboelastometry (ROTEM) to monitor haemostatic treatment versus usual care in adults or children with bleeding. Cochrane Database Syst. Rev..

[55] Mallaiah S., Barclay P., Harrod I., Chevannes C., Bhalla A. (2015). Introduction of an algorithm for ROTEM-guided fibrinogen concentrate administration in major obstetric haemorrhage. Anaesthesia.

[56] Pacagnella R.C., Souza J.P., Durocher J., Perel P., Blum J., Winikoff B., Gülmezoglu A.M. (2013). A systematic review of the relationship between blood loss and clinical signs. PLoS ONE.

[57] Qiu X., Nair M.G., Jaroszewski L., Godzik A. (2024). Deciphering Abnormal Platelet Subpopulations in COVID-19, Sepsis and Systemic Lupus Erythematosus through Machine Learning and Single-Cell Transcriptomics. Int. J. Mol. Sci..

[58] Rajkomar A., Dean J., Kohane I. (2019). Machine learning in medicine. N. Engl. J. Med..

[59] Christodoulou E., Ma J., Collins G.S., Steyerberg E.W., Verbakel J.Y., Van Calster B. (2019). A systematic review shows no performance benefit of machine learning over logistic regression for clinical prediction models. J. Clin. Epidemiol..

[60] Obermeyer Z., Emanuel E.J. (2016). Predicting the future—Big data, machine learning, and clinical medicine. N. Engl. J. Med..

[61] Shortliffe E.H., Sepúlveda M.J. (2018). Clinical decision support in the era of artificial intelligence. JAMA.

[62] Wada H., Thachil J., Di Nisio M., Mathew P., Kurosawa S., Gando S., Kim H.K., Nielsen J.D., Dempfle C.E., Levi M. (2013). Guidance for diagnosis and treatment of disseminated intravascular coagulation from harmonization of the recommendations from three guidelines. J. Thromb. Haemost..

[63] Debray T.P.A., Vergouwe Y., Koffijberg H., Nieboer D., Steyerberg E.W., Moons K.G. (2015). A new framework to enhance the interpretation of external validation studies of clinical prediction models. J. Clin. Epidemiol..

[64] Hemming K., Haines T.P., Chilton P.J., Girling A.J., Lilford R.J. (2015). The stepped wedge cluster randomised trial: Rationale, design, analysis, and reporting. BMJ.

[65] Ancker J.S., Edwards A., Nosal S., Hauser D., Mauer E., Kaushal R., HITEC Investigators (2017). Effects of workload, work complexity, and repeated alerts on alert fatigue in a clinical decision support system. BMC Med. Inform. Decis. Mak..

[66] Sanders G.D., Neumann P.J., Basu A., Brock D.W., Feeny D., Krahn M., Kuntz K.M., Meltzer D.O., Owens D.K., Prosser L.A. (2016). Recommendations for conduct, methodological practices, and reporting of cost-effectiveness analyses: Second panel on cost-effectiveness in health and medicine. JAMA.

