# Supplementary material for: PAD Score: A Clinical Prediction Tool for Disseminated Intravascular Coagulation in Placental Abruption
Source: J Clin Med. 2026 May 5;15(9):3524. doi: 10.3390/jcm15093524 (PMC13164300; doi:10.3390/jcm15093524)
Supplement: Supplementary file 1 [file jcm-15-03524-s001.zip › jcm-4188871-supplementary.pdf]

Supplementary Materials — PAD Score (jcm-4188871)

Supplementary Table S1. Completed TRIPOD 22-item checklist for development and internal validation of the PAD Score.

Section / Topic	Item	Checklist item	Reported on
Title & Abstract	1	Identify the study as developing and/or validating a multivariable prediction model, target population, outcome predicted	Title; Keywords
	2	Provide a summary of objectives, study design, setting, participants, sample size, predictors, outcome, methods, results, and conclusions	Abstract
Introduction	3a	Explain medical context and rationale for developing/validating the model	Section 1, paras 1–4
	3b	Specify the objectives, including whether the study describes the development or validation of the model, or both	Section 1, final paragraph
Methods — Source of data	4a	Describe study design or source of data (e.g., RCT, cohort, registry)	Section 2.1
	4b	Specify the key study dates, including start of accrual, end of accrual, and, if applicable, end of follow-up	Section 2.1 (1 Jan 2019 – 31 Dec 2024)
Methods — Participants	5a	Specify key elements of the study setting including number and location of centres	Section 2.1
	5b	Describe eligibility criteria for participants	Section 2.2
	5c	Give details of treatments received, if relevant	Section 2.2
Methods — Outcome	6a	Clearly define the outcome that is predicted by the prediction model, including how and when assessed	Section 2.5
	6b	Report any actions to blind assessment of the outcome to be predicted	Section 2.5 (laboratory-based outcome)
Methods — Predictors	7a	Clearly define all predictors used in developing or validating the multivariable prediction model, including how and when they were measured	Section 2.4
	7b	Report any actions to blind assessment of predictors for the outcome and other predictors	Section 2.4
Methods — Sample size	8	Explain how the study size was arrived at	Section 2.3
Methods — Missing data	9	Describe how missing data were handled (e.g., complete-case analysis, single imputation, multiple imputation) with details of any imputation method	Section 2.6; Supp Table S5
Methods — Statistical analysis	10a	Describe how predictors were handled in the analyses	Section 2.6
	10b	Specify type of model, all model-building procedures (including any predictor selection), and method for internal validation	Section 2.6
	10d	Specify all measures used to assess model performance and, if relevant, to compare multiple models	Section 2.6

Section / Topic	Item	Checklist item	Reported on
Methods — Risk groups	11	Provide details on how risk groups were created, if done	Section 2.6; Table 7
Results — Participants	13a	Describe the flow of participants through the study, including the number of participants with and without the outcome and, if applicable, a summary of the follow-up time	Section 3.1
	13b	Describe the characteristics of the participants (basic demographics, clinical features, available predictors), including the number of participants with missing data for predictors and outcome	Section 3.1; Table 2
Results — Model development	14a	Specify the number of participants and outcome events in each analysis	Section 3.1
	14b	If done, report the unadjusted association between each candidate predictor and outcome	Table 3
Results — Model specification	15a	Present the full prediction model to allow predictions for individuals (i.e., all regression coefficients, and model intercept or baseline survival at a given time point)	Table 4
	15b	Explain how to use the prediction model	Section 4.4; Table 6–8
Results — Model performance	16	Report performance measures (with CIs) for the prediction model	Table 5; Figures 1–4
Results — Model-updating	17	If done, report the results from any model updating (i.e., model specification, model performance)	Not applicable
Discussion — Limitations	18	Discuss any limitations of the study (such as non-representative sample, few events per predictor, missing data)	Section 4.7
Discussion — Interpretation	19b	Give an overall interpretation of the results, considering objectives, limitations, and results from similar studies, and other relevant evidence	Section 4.1–4.5
Discussion — Implications	20	Discuss the potential clinical use of the model and implications for future research	Section 4.4; 4.8
Other — Supplementary	21	Provide information about the availability of supplementary resources, such as study protocol, Web calculator, and data sets	Data Availability Statement
Other — Funding	22	Give the source of funding and the role of the funders for the present study	Funding statement

Checklist items are adapted from: Collins GS, Reitsma JB, Altman DG, Moons KGM. Transparent Reporting of a multivariable prediction model for Individual Prognosis Or Diagnosis (TRIPOD): the TRIPOD statement. BMJ. 2015;350:g7594.

Supplementary Table S2. Diagnostic confirmation of placental abruption and categorical distribution of placental separation percentage, stratified by DIC status.

Part A. Modalities of diagnostic confirmation.

Confirmation modality	Full cohort (n=237) n (%)	No DIC (n=183) n (%)	DIC (n=54) n (%)	p-value
Ultrasonographic confirmation only	85 (35.9)	66 (36.1)	19 (35.2)	0.91
Histopathological confirmation only	32 (13.5)	26 (14.2)	6 (11.1)	0.55
Both ultrasonographic + histopathological	120 (50.6)	91 (49.7)	29 (53.7)	0.61

Part B. Categorical distribution of placental separation percentage.

Placental separation category	Full cohort n (%)	No DIC n (%)	DIC n (%)
< 10 %	31 (13.1)	29 (15.8)	2 (3.7)
10 – 19 %	54 (22.8)	50 (27.3)	4 (7.4)
20 – 39 %	105 (44.3)	87 (47.5)	18 (33.3)
≥ 40 %	47 (19.8)	17 (9.3)	30 (55.6)

p-values compare the DIC-positive and DIC-negative groups (χ^2 or Fisher exact test as appropriate). Separation-category thresholds correspond to the PAD Score integer-point strata. DIC, disseminated intravascular coagulation; PAD, Placental Abruption DIC.

Supplementary Table S3. Robustness of overt DIC outcome classification across alternative scoring systems.

**Scoring definition**	**n DIC-positive (%)**	**Cohen's κ vs primary (95% CI)**	**Percent agreement**	**PABAK**
Original ISTH ≥ 5 (primary)	54 (22.8)	reference	100.0%	reference
Original ISTH ≥ 6	54 (22.8)	**κ not estimable (perfect agreement; no discordant pairs; see Table S6)**	100.0%	1.00
Erez pregnancy-modified ≥ 26	52 (21.9)	0.93 (0.87–0.99)	97.5%	0.95
Kobayashi obstetric ≥ 8	58 (24.5)	0.86 (0.78–0.94)	94.9%	0.90

Agreement statistics are computed relative to the primary outcome classification (original ISTH ≥ 5). PABAK, prevalence-adjusted bias-adjusted kappa.

Supplementary Table S4. Likelihood-ratio tests for nonlinearity of continuous predictors in the PAD Score multivariable model.

Predictor	Knots	LR χ^2	df	p-value
Fibrinogen (mg/dL)	3	1.84	1	0.175
Fibrinogen (mg/dL)	4	3.12	2	0.210
Fibrinogen (mg/dL)	5	4.58	3	0.205
Shock index	3	0.96	1	0.327
Shock index	4	2.41	2	0.300
Shock index	5	3.74	3	0.291
Platelet ($\times 10^9$/L)	3	1.26	1	0.262
Platelet ($\times 10^9$/L)	4	2.88	2	0.236
Platelet ($\times 10^9$/L)	5	4.12	3	0.249
Placental separation (%)	3	2.07	1	0.150
Placental separation (%)	4	3.65	2	0.161
Placental separation (%)	5	5.41	3	0.144

Restricted cubic splines were fitted for each continuous predictor with 3, 4, and 5 knots. Likelihood-ratio tests compare the nonlinear model against the linear specification. All p-values exceed 0.10, supporting use of linear terms in the final model. Analyses performed in R v4.3.2 using the rms package (version 6.7-1). LR, likelihood ratio; df, degrees of freedom

Supplementary Table S5. Pattern of missing data and multiple imputation strategy for candidate predictors (N = 237).

**Variable**	**Missing, n (%)**	**Mechanism (pattern)**	**Handling**
Fibrinogen	10 (4.2)	MCAR	MICE (PMM)
D-dimer	17 (7.2)	MCAR	MICE (PMM)
Shock index	3 (1.3)	MCAR	MICE (PMM)
Placental separation, %	0 (0.0)	Complete	—
Platelet count	0 (0.0)	Complete	—
Hemoglobin at admission	0 (0.0)	Complete	—
Prothrombin time	5 (2.1)	MCAR	MICE (PMM)
Activated partial thromboplastin time	5 (2.1)	MCAR	MICE (PMM)
International normalized ratio	5 (2.1)	MCAR	MICE (PMM)
Liver function tests (AST / ALT)	11 (4.6)	MCAR	MICE (PMM)
Creatinine	2 (0.8)	MCAR	MICE (PMM)
Chronic hypertension / preeclampsia	0 (0.0)	Complete	—
Prior placental abruption	0 (0.0)	Complete	—
Body mass index	6 (2.5)	MCAR	MICE (PMM)
Smoking during pregnancy	8 (3.4)	MCAR	MICE (logistic)
**Overall Little's MCAR test**	—	χ^2 = 102.4, df = 96, p = 0.34	—

MICE = multiple imputation by chained equations; PMM = predictive mean matching; MCAR; missing completely at random; df, degrees of freedom.

Supplementary Table S6. Frequency distribution of the original ISTH overt DIC score (0–11), stratified by final DIC classification (N = 237).

ISTH Score	DIC-negative (n)	DIC-positive (n)	Total (n)
0	50	0	50
1	48	0	48
2	35	0	35
3	30	0	30
4	20	0	20
**5**	**0**	**0**	**0**
6	0	15	15
7	0	14	14
8	0	10	10
9	0	8	8
10	0	5	5
11	0	2	2
**Total**	**183**	**54**	**237**

The frequency distribution is bimodal: DIC-negative cases cluster at ISTH scores 0–4 (range 0–4, median 1) and DIC-positive cases cluster at ISTH scores 6–11 (range 6–11, median 7, IQR 6–9). No patient had an ISTH score of exactly 5. This complete separation at the cut-point is the mechanism underlying the perfect inter-algorithmic agreement reported in Table S3 (original ISTH ≥ 5 vs ≥ 6), and explains why the primary outcome classification does not depend on the choice of threshold within the 5–6 point region. The finding reflects the characteristic clinical pattern of placental abruption-associated DIC, in which laboratory derangements are typically so pronounced at the time of presentation that they fall well in excess of the diagnostic thresholds of any candidate scoring system.

Supplementary Table S7. Consolidated sensitivity analyses for discrimination and calibration.

Sensitivity analysis	Dataset / approach	AUC (95% CI)	Calibration slope	ΔAUC vs primary
Primary (MICE, 20 imputations, Rubin's rules)	Full cohort (n = 237)	0.916 (0.882–0.954)	0.96	—
Complete-case analysis	n = 207	0.909 (0.869–0.949)	0.94	−0.007
Single imputation (median / mode)	n = 237	0.913 (0.878–0.948)	0.95	−0.003
Firth-penalized logistic regression	n = 237	0.914 (0.880–0.948)	0.95	−0.002
Excluding patients transferred from external facility	n = 148	0.902 (0.854–0.950)	0.92	−0.014
Four-variable model (preeclampsia excluded)	n = 237	0.892 (0.852–0.932)	0.93	−0.024
Alternative cut-offs (0–3 / 4–7 / ≥ 8)	n = 237	0.916 (0.882–0.954)	0.96	0.000
Reduced model (fibrinogen + platelet count excluded)	n = 237	0.842 (0.782–0.902)	0.90	−0.074
Composite clinical outcome (MTP / hysterectomy / ICU ≥ 48 h / death)	n = 237	0.801 (0.739–0.863)	0.88	−0.115
Pathology-confirmed subgroup	n = 152	0.887 (0.840–0.934)	0.93	−0.029

AUC, area under the receiver operating characteristic curve; CI, confidence interval; MICE, multiple imputation by chained equations; MTP, massive transfusion protocol; ICU, intensive care unit.

Supplementary Table S8. Comparative discrimination against established obstetric DIC scoring systems.

DIC scoring system	AUC (95% CI)	ΔAUC vs PAD	DeLong p	IDI	Continuous NRI
PAD Score (present study)	0.916 (0.882–0.954)	reference	—	—	—
ISTH overt DIC score (applied predictively at admission)	0.812 (0.751–0.873)	−0.104	<0.001	0.182	0.354
Erez pregnancy-modified DIC score	0.848 (0.792–0.904)	−0.068	0.002	0.124	0.267
Kobayashi obstetric DIC score	0.793 (0.729–0.857)	−0.123	<0.001	0.196	0.388

ΔAUC, IDI (integrated discrimination improvement), and continuous NRI (net reclassification improvement) are computed versus the primary PAD Score. DeLong p values correspond to the paired ROC comparison against PAD. AUC, area under the receiver operating characteristic curve; CI, confidence interval; ROC, receiver operating characteristic.

Supplementary Figure S1. Calibration plot of the full PAD model for prediction of overt disseminated intravascular coagulation.

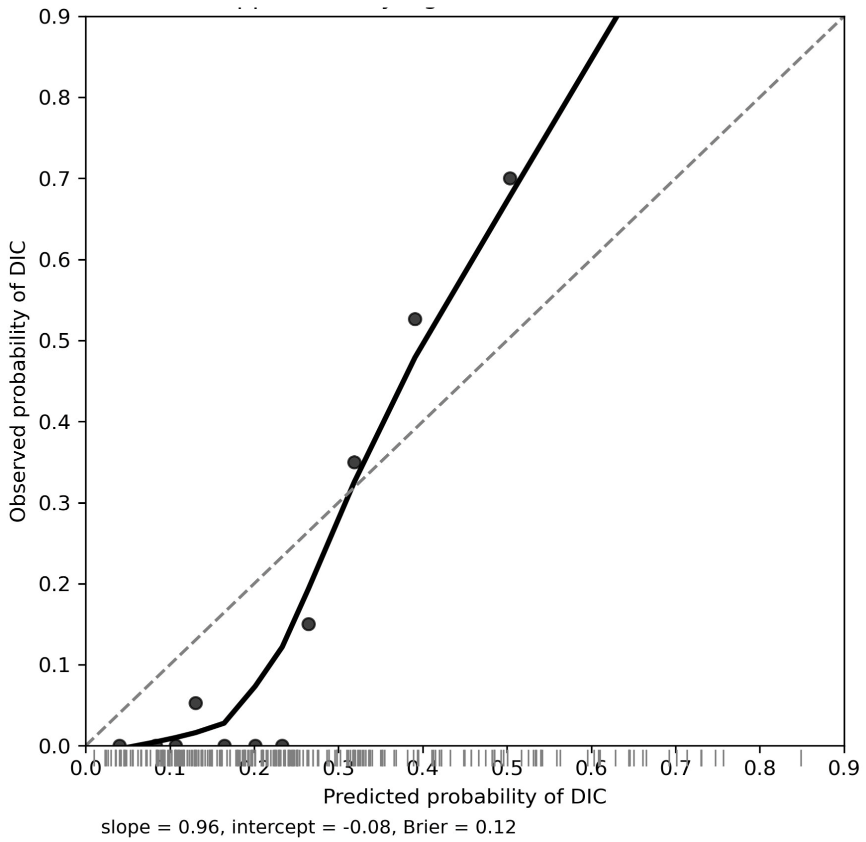

The plot shows LOESS-smoothed observed versus predicted probabilities of overt DIC across the range of model-estimated risk. The gray dashed line represents the 45° line of perfect calibration. Rug marks along the x-axis indicate the distribution of predicted risks in the study cohort. Model calibration metrics were as follows: calibration slope = 0.96, calibration intercept = −0.08, and Brier score = 0.12.

Supplementary Figure S2. Distribution of placental separation percentage in the analytic cohort, overlaid by DIC status.

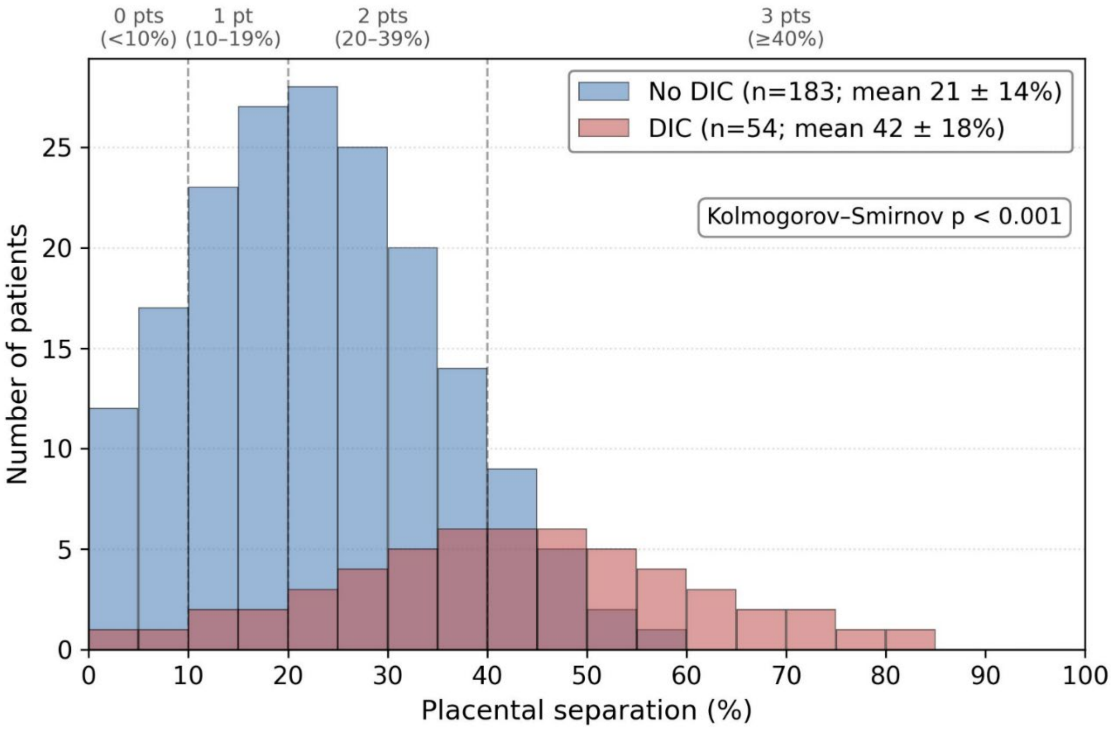

Overlaid histogram of ultrasonographically estimated placental separation percentage in the 237-patient analytic cohort, stratified by subsequent development of overt DIC within 24 h of admission. Descriptive statistics: DIC-positive 42% (SD 18); DIC-negative 21% (SD 14); Kolmogorov–Smirnov $p < 0.001$. Vertical reference lines at the PAD Score point-category thresholds (10%, 20%, 40%) are provided for interpretive context.
